# Patterns in metabolite profile are associated with risk of more aggressive prostate cancer: A prospective study of 3,057 matched case–control sets from EPIC

**DOI:** 10.1002/ijc.32314

**Published:** 2019-04-29

**Authors:** Julie A. Schmidt, Georgina K. Fensom, Sabina Rinaldi, Augustin Scalbert, Paul N. Appleby, David Achaintre, Audrey Gicquiau, Marc J. Gunter, Pietro Ferrari, Rudolf Kaaks, Tilman Kühn, Heiner Boeing, Antonia Trichopoulou, Anna Karakatsani, Eleni Peppa, Domenico Palli, Sabina Sieri, Rosario Tumino, Bas Bueno‐de‐Mesquita, Antonio Agudo, Maria‐Jose Sánchez, María‐Dolores Chirlaque, Eva Ardanaz, Nerea Larrañaga, Aurora Perez‐Cornago, Nada Assi, Elio Riboli, Konstantinos K. Tsilidis, Timothy J. Key, Ruth C. Travis

**Affiliations:** ^1^ Cancer Epidemiology Unit, Nuffield Department of Population Health University of Oxford Oxford United Kingdom; ^2^ International Agency for Research on Cancer Lyon France; ^3^ Division of Cancer Epidemiology German Cancer Research Center (DKFZ) Heidelberg Germany; ^4^ Department of Epidemiology German Institute of Human Nutrition (DIfE) Potsdam‐Rehbrücke Nuthetal Germany; ^5^ Hellenic Health Foundation Athens Greece; ^6^ 2nd Pulmonary Medicine Department School of Medicine, National and Kapodistrian University of Athens, “ATTIKON” University Hospital Haidari Greece; ^7^ Cancer Risk Factors and Life‐Style Epidemiology Unit Institute for Cancer Research, Prevention and Clinical Network (ISPRO) Florence Italy; ^8^ Epidemiology and Prevention Unit Fondazione IRCCS Istituto Nazionale dei Tumori Milano Italy; ^9^ Cancer Registry and Histopathology Department “Civic ‐ M.P.Arezzo" Hospital, Azienda Sanitaria Provinciale Di Ragusa (ASP) Ragusa Italy; ^10^ Department for Determinants of Chronic Diseases (DCD) National Institute for Public Health and the Environment (RIVM) Bilthoven The Netherlands; ^11^ Department of Gastroenterology and Hepatology University Medical Centre Utrecht The Netherlands; ^12^ Department of Epidemiology and Biostatistics School of Public Health, Imperial College London London United Kingdom; ^13^ Department of Social & Preventive Medicine, Faculty of Medicine University of Malaya Kuala Lumpur Malaysia; ^14^ Unit of Nutrition and Cancer, Cancer Epidemiology Research Program Catalan Institute of Oncology‐IDIBELL, L'Hospitalet de Llobregat Barcelona Spain; ^15^ CIBER in Epidemiology and Public Health (CIBERESP) Madrid Spain; ^16^ Escuela Andaluza de Salud Pública, Instituto de Investigación Biosanitaria ibs.GRANADA Hospitales Universitarios de Granada/Universidad de Granada Granada Spain; ^17^ Department of Epidemiology Regional Health Council, IMIB‐Arrixaca Murcia Spain; ^18^ Department of Health and Social Sciences Murcia University Murcia Spain; ^19^ Navarra Public Health Institute Pamplona Spain; ^20^ IdiSNA, Navarra Institute for Health Research Pamplona Spain; ^21^ Basque Regional Health Department Public Health Division of Gipuzkoa‐BIODONOSTIA San Sebastian Spain; ^22^ Department of Hygiene and Epidemiology University of Ioannina School of Medicine Ioannina Greece

**Keywords:** epidemiology, metabolomics, prostate cancer risk, treelet transform

## Abstract

Metabolomics may reveal novel insights into the etiology of prostate cancer, for which few risk factors are established. We investigated the association between patterns in baseline plasma metabolite profile and subsequent prostate cancer risk, using data from 3,057 matched case–control sets from the European Prospective Investigation into Cancer and Nutrition (EPIC). We measured 119 metabolite concentrations in plasma samples, collected on average 9.4 years before diagnosis, by mass spectrometry (Absolute*IDQ* p180 Kit, Biocrates Life Sciences AG). Metabolite patterns were identified using treelet transform, a statistical method for identification of groups of correlated metabolites. Associations of metabolite patterns with prostate cancer risk (OR_1SD_) were estimated by conditional logistic regression. Supplementary analyses were conducted for metabolite patterns derived using principal component analysis and for individual metabolites. Men with metabolite profiles characterized by higher concentrations of either phosphatidylcholines or hydroxysphingomyelins (OR_1SD_ = 0.77, 95% confidence interval 0.66–0.89), acylcarnitines C18:1 and C18:2, glutamate, ornithine and taurine (OR_1SD_ = 0.72, 0.57–0.90), or lysophosphatidylcholines (OR_1SD_ = 0.81, 0.69–0.95) had lower risk of advanced stage prostate cancer at diagnosis, with no evidence of heterogeneity by follow‐up time. Similar associations were observed for the two former patterns with aggressive disease risk (the more aggressive subset of advanced stage), while the latter pattern was inversely related to risk of prostate cancer death (OR_1SD_ = 0.77, 0.61–0.96). No associations were observed for prostate cancer overall or less aggressive tumor subtypes. In conclusion, metabolite patterns may be related to lower risk of more aggressive prostate tumors and prostate cancer death, and might be relevant to etiology of advanced stage prostate cancer.

## Abstract 2

**What's new?**

This prospective study is the largest investigation of metabolite profile and prostate cancer risk, to date. We found that patterns in plasma metabolite profile (characterized by higher concentrations of phosphatidylcholines and hydroxysphingomyelins; specific acylcarnitines, amino acids and a biogenic amine; and lysophosphatidylcholines, respectively) were associated with subsequent lower risk of more aggressive tumor subtypes and prostate cancer death. Moreover, the results suggest that metabolite profile may be relevant to the etiology of advanced stage disease.

**Abbreviations**
ATBC: Alpha‐Tocopherol, Beta‐Carotene Cancer Prevention Study
CI: confidence interval
EPIC: European Prospective Investigation into Cancer and Nutrition
IARC: International Agency for Research on Cancer
LysoPC: lysophosphatidylcholines
OR_1SD_: odds ratio for a one standard deviation increase in treelet component score
PC aa: diacyl phosphatidylcholines
PC ae: acyl‐alkyl phosphatidylcholines
PCA: principal component analysis
*r*: correlation coefficient
SD: standard deviation
SM (OH): hydroxysphingomyelins
SM: sphingomyelins
TNM: tumor‐node‐metastasis

## Introduction

Prostate cancer is the second most commonly diagnosed cancer in men worldwide, but few risk factors have been established.^1^, ^2^ Using metabolomics, which measures large numbers of small molecules in body fluids reflecting internal (the genome, epigenome, transcriptome and proteome) and external factors (diet, lifestyle, environment and gut microbiota),^3^, ^4^, ^5^ may help to identify novel risk factors for prostate cancer. Previously, relatively small prospective studies (*n*_cases_ = 74–1,077), including our study in the European Prospective Investigation into Cancer and Nutrition (EPIC),^6^ showed that men with higher circulating concentrations of lipids and/or energy‐related metabolites might have lower risk of prostate cancer, especially more aggressive tumor subtypes.^6^, ^7^, ^8^, ^9^ Moreover, a prospective study of lethal prostate cancer reported associations with metabolites in redox (inverse), dipeptide (positive), pyrimidine (mostly positive) and gamma‐glutamyl amino acid (positively) pathways (*n*_deaths_ = 523).^10^ These studies mostly had limited power and have primarily investigated individual metabolites, which do not capture patterns in the metabolite profile reflecting correlations among metabolites^11^; such patterns might represent metabolites which are part of the same metabolic pathway^12^ or originating from the same dietary sources.^13^ To overcome this and the issue of multiple testing, dimension‐reduction methods such as treelet transform can be applied. Treelet transform combines features from principal component analysis (PCA) and cluster analysis to describe latent patterns in the data, which often are easier to interpret than those of PCA because metabolites irrelevant to the pattern are omitted. Treelet transform has for example been used to successfully describe patterns in serum metabolite profile^12^ and to identify patterns in plasma fatty acids which were related to subsequent prostate cancer.^14^

We report an extension of our previous case–control study nested in EPIC (*n*_case_ = 1,077),^6^ now including 3,057 matched case–control sets, which, to the best of our knowledge, is the largest study to date of metabolites and prostate cancer risk. We aimed to identify patterns in plasma metabolite profiles in men without prostate cancer using treelet transform and to estimate the prospective associations between patterns and subsequent risk of prostate cancer overall, and by tumor characteristics and time to diagnosis, and risk of prostate cancer death.

## Materials and Methods

### Study population

The EPIC study includes 153,400 men recruited between 1992 and 2000 from 19 centers in eight countries (Denmark, Germany, Greece, Italy, Netherlands, Spain, Sweden and United Kingdom).^15^ At recruitment, detailed information was collected on diet and lifestyle, and 139,600 men gave a blood sample. For the current study, men were eligible if they had blood stored at the central biobank at the International Agency of Research on Cancer, Lyon, France (IARC; centers in Germany, Greece, Italy, Netherlands, Spain and United Kingdom), the date of blood collection was known, and if no cancer (except non‐melanoma skin cancer) had been diagnosed at the time of blood collection. All participants gave written informed consent and the study was approved by ethical committees of IARC and the participating centers.

### Follow‐up and case and control selection

In most study centers, information on cancer incidence, tumor subtypes and vital status was obtained via record linkage to regional and national cancer registries. However, in Germany and Greece, a combination of methods was used, including health insurance records, cancer and pathology registries and active follow‐up; self‐reported incident cancers were verified through medical records.

Cases were men diagnosed with prostate cancer (defined as code C61 in the 10th revision of the International Statistical Classification of Diseases and Related Health Problems [ICD‐10]) after blood collection and before the end of follow‐up in August 2014. Each case was matched on the study center, length of follow‐up and age (±6 months), time of day (±1 hr) and fasting status (<3, 3–6, >6 hr) at blood collection to one control participant, selected randomly among male cohort participants who were alive and free of cancer (except non‐melanoma skin cancer) at the time of diagnosis of the case; an incidence density sampling procedure was used.

Prostate cancer subtypes were categorized based on the tumor‐node‐metastasis (TNM) system and histological grade as follows. Localized (≤T_2_ and N_0/x_ and M_0_, or stage coded as localized, *n* = 1,306), advanced (T_3–4_ and/or N_1–3_ and/or M_1_, or coded as advanced, *n* = 580), non‐aggressive (≤T_3_ and N_0/x_ and M_0_, *n* = 1,519), aggressive (a subset of advanced stage disease defined as T_4_ and/or N_1–3_ and/or M_1_, *n* = 367), low‐intermediate grade (Gleason score <8 or coded as well, moderately or poorly differentiated tumors, *n* = 2,157) and high grade (Gleason score ≥8 or coded as undifferentiated tumors, *n* = 317)_._ Death from prostate cancer was defined as prostate cancer listed as the underlying cause of death on the death certificate during follow‐up (*n* = 326).

### Blood collection and laboratory analysis

A standardized protocol for blood collection and processing was followed and fasting was not required (details published elsewhere^15^). All plasma samples (citrate anticoagulant) were assayed at IARC, using the Absolute*IDQ* p180 Kit (Biocrates Life Sciences AG, Innsbruck, Austria) and following the procedure recommended by the vendor. Samples from matched case–control sets were assayed in the same analytical batch along with quality control samples from pooled plasma; laboratory personnel was blinded to sample category, that is, case, study control or quality control. A triple quadrupole mass spectrometer (Triple Quad 4500; AB Sciex, Framingham, MA) was used to quantify 148 metabolites. After exclusions of metabolites and men, 3,057 matched case–control sets had data for 119 metabolites (details in Supporting Information Methods and Table S1). The metabolites comprised 8 acylcarnitines, 21 amino acids, 5 biogenic amines, 72 phosphatidylcholines (lysophosphatidylcholines [lysoPC, *n* = 8], diacyl phosphatidylcholines [PC aa, *n* = 31] and acyl‐alkyl phosphatidylcholines [PC ae, *n* = 33]), hexose and 12 sphingomyelins (denoted hydroxysphingomyelins [SM (OH), *n* = 5] and sphingomyelins [SM, *n* = 7]). See Supporting Information Methods for metabolite nomenclature.

### Statistical analyses

Patterns in metabolite profile were identified using treelet transform as described by Gorst‐Rasmussen *et al*.^16^, ^17^ In brief, treelet transform is a data‐driven linear dimension‐reduction method, which produces orthogonal components (vectors) from variables, for example, metabolites, based on their correlation or covariance structure. The numeric size of a variable within the component is called the loading and quantifies the contribution of each variable (here metabolite) to the component. A key aspect of treelet transform is its sparsity feature whereby loadings for some variables irrelevant to a given component are set to zero; this simplifies the interpretation of the patterns.

**Figure 1:**
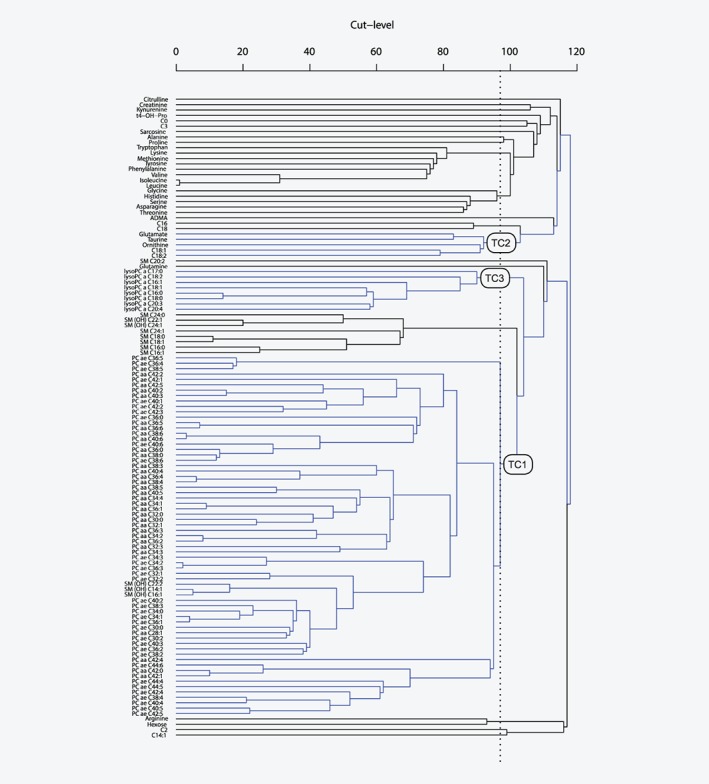
Cluster tree from treelet transform on 119 metabolite concentrations. The dotted line indicates the cut‐level at 97. Blue lines indicate joining of correlated metabolites which belong to the three retained treelet components (TC). *n* = 3,057 control participants from EPIC.

In treelet transform, the two most closely correlated metabolites are joined together by local PCA and the two original variables are replaced with the first principal component. This procedure is repeated until all variables are joined into one and the structure of the data is visualized by a cluster tree (Fig. 1). To obtain the treelet components the cluster tree is cut at a level providing a good trade‐off between maximizing both the variance explained (higher cut‐level) and the sparsity of the components (lower cut‐level); the optimal cut‐level for a given number of retained components is determined by data‐driven cross‐validation. Finally, for each retained component, a score variable is calculated as the linear combination of a participant's concentrations of the metabolites weighted by the loadings. The score reflects how similar a participant's metabolite profile is to the metabolite pattern.

In the current study, treelet transform was based on control participants only using the covariance matrix of logarithmically transformed metabolite concentrations. To inform the choice of number of factors to retain and the corresponding cut‐level, we ran treelet transform for different numbers of retained components (1–10), identified the optimal cut‐level for each (ranging from 97 to 104) using cross‐validation, inspected scree plots (Supporting Information Fig. S1)^17^ and assessed pattern interpretability.^18^ The final treelet transform was then computed specifying the number of components to retain and the lowest optimal cut‐level. Finally, component scores were calculated for all participants. A sensitivity analysis of the cut‐level and a stability assessment of the treelet components were performed.

We used conditional logistic regression to estimate risk of prostate cancer for quintiles of component scores and per one SD increase in component score. The analysis was conditioned on the matching variables and further adjusted for exact age at blood collection (continuously), baseline values of body mass index (quartiles; unknown), smoking (never, past, current and unknown), alcohol intake (<10, 10–19, 20–39 and ≥40 g of alcohol/day; unknown), attained education level (primary, secondary, degree level and unknown) and marital status (married or cohabiting, not married or cohabiting, and unknown).

Similar conditional logistic regression models were fitted for subgroup analyses by tumor subtypes (localized/advanced, non‐aggressive/aggressive and high/low‐intermediate grade), and likelihood ratio tests for heterogeneity in the association by subtypes were conducted. To assess potential presence of reverse causation in the associations of metabolite patterns with risk of more aggressive prostate cancer stages, the analyses for advanced and aggressive prostate cancer were further stratified by time to diagnosis (≤10/>10 years). In the analysis of risk of prostate cancer death, matched sets in which the control died, emigrated or was lost to follow‐up before the case died were excluded (*n* = 29).

In a sensitivity analysis, the models examining the association between any treelet component and risk were additionally adjusted for any other treelet components (as continuous variables) with a Pearson correlation higher than 0.2 in absolute value with the treelet component of interest.

As a secondary analysis, we repeated the analysis of treelet components and risk of overall prostate cancer in the subset of participants, who were not included in our previously published analysis (*n*_case_ = 2,018).^6^

For comparison with the treelet transform method, we additionally identified metabolite patterns using PCA and assessed their association with risk of prostate cancer, in the entire study population.

For the purpose of comparison with previous studies, we also computed the associations of each individual metabolite and risk of prostate cancer, while accounting for multiple testing. This analysis was done for the full data set (*n*_case_ = 3,057), and separately for the data not previously published^6^ (*n*_case_ = 2,018).

All tests of statistical significance were two‐sided, and 0.05 was considered as the nominal level for statistical significance in analyses of metabolite patterns and prostate cancer risk. All analyses were conducted in Stata Statistical Software Package, version 15 (Stata Corporation, College Station, TX). The “tt” add‐on for STATA is available from http://www.gorst.dk. See Supporting Information Methods for further details of statistical analyses.

## Results

At blood collection, participants were on average 58 years old (SD = 7.3 years) and cases were diagnosed on average 9.4 years later. There were no marked differences between baseline characteristics of the cases and controls (Table 1).

**Table 1:** Characteristics of prostate cancer cases and controls in EPIC

Characteristic	Cases, *n* = 3,057	Controls, *n* = 3,057
Age at blood collection, years (SD)	58.0 (7.3)	58.0 (7.3)
Height, cm (SD)^1^	172.2 (7.0)	172.4 (7.1)
Body Mass Index, kg/m^2^ (SD)^1^	27.2 (3.4)	27.2 (3.5)
Smoking, *n* (%)^1^		
Never	1,031 (34.3)	938 (31.1)
Former	1,276 (42.4)	1,316 (43.6)
Current	703 (23.4)	765 (25.3)
Alcohol consumption, *n* (%)^1^		
<10 g/day	1,271 (41.7)	1,272 (41.6)
10–19 g/day	577 (18.9)	577 (18.9)
20–40 g/day	632 (20.7)	674 (22.1)
≥40 g/day	566 (18.6)	533 (17.4)
Physical activity, *n* (%)^1^		
Inactive	710 (23.7)	721 (24.1)
Moderately inactive	986 (33.0)	985 (32.9)
Moderately active	716 (23.9)	698 (23.3)
Active	579 (19.4)	592 (19.8)
Marital status, *n* (%)^1^		
Married or cohabiting	2,051 (88.5)	2,063 (88.9)
Not married or cohabiting	267 (11.5)	257 (11.1)
Educational attainment, *n* (%)^1^		
Primary or equivalent	1,181 (40.6)	1,195 (40.9)
Secondary	1,001 (34.4)	1,026 (35.1)
Degree	727 (25.0)	704 (24.1)
Cases only		
Age at diagnosis, years (SD)^1^	67.4 (6.9)	–
Time to diagnosis, years (SD)^1^^,^ 2	9.4 (4.2)	
Stage, *n* (%)^1^^,^ 3		
Localized	1,306 (69.2)	–
Advanced	580 (30.8)	–
Stage (aggressiveness), *n* (%)^1^^,^ 3		
Non‐aggressive	1,519 (80.5)	–
Aggressive	367 (19.5)	–
Grade, *n* (%)^1^^,^ 4		
Low‐intermediate grade	2,157 (87.2)	–
High grade	317 (12.8)	–
Death from prostate cancer, *n* (%)^1^^,^ 5	297 (9.7)	–

1 Unknown values for some participants; the calculations of percentages exclude missing values.

2 Time between blood collection and diagnosis.

3 The tumor‐node‐metastasis (TNM) system was used to categorize stages of prostate cancer; localized: ≤T_2_ and N_0/x_ and M_0_, or coded as localized; advanced: T_3–4_ and/or N_1–3_ and/or M_1_, or coded as advanced; non‐aggressive: ≤T_3_ and N_0/x_ and M_0;_ and aggressive: T_4_ and/or N_1–3_ and/or M_1_. All categories are not mutually exclusive as aggressive is a subset of advanced stage, so numbers do not add up; percentages were calculated separately for localized and advance, and for non‐aggressive and aggressive.

4 Gleason score <8 or coded as well, moderately or poorly differentiated for low‐intermediate grade and Gleason score ≥8 or coded as undifferentiated for high grade.

5 Death from prostate cancer (prostate cancer listed as the underlying cause of death on the death certificate) during follow‐up; 326 died from prostate cancer, but 29 were excluded from further analysis as their matched control either died, emigrated or was lost to follow‐up before they died.

### Treelet patterns in metabolite profile

**Figure 2:**
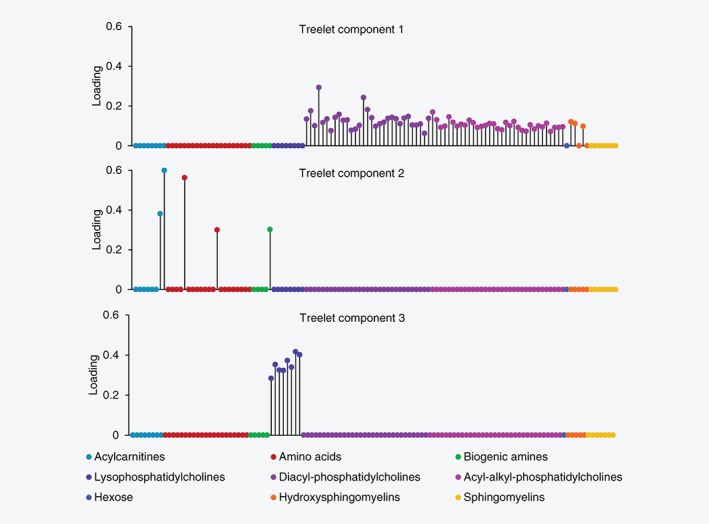
Loading plots for treelet components 1, 2 and 3 from treelet transform on 119 metabolite concentrations, using cut‐level 97. The loading is the numeric size of a metabolite within the component, and it quantifies the contribution of each metabolite to the component. *n* = 3,057 control participants from EPIC.

After inspection of scree plots from treelet transform (Supporting Information Fig. S1) and cross‐validation results, we retained three patterns at cut‐level 97; the hierarchical grouping of metabolites is shown in Figure 1. Together, the three patterns explained 31.4% of the total variation in metabolite concentrations, with the first to the third pattern (treelet components 1–3) accounting for 21.5, 5.2 and 4.7%, respectively (Supporting Information Table S2). Treelet component 1 was characterized by positive loadings on all diacyl and acyl‐alkyl phosphatidylcholines and three hydroxysphingomyelins (Fig. 2; Supporting Information Table S2). Treelet component 2 was characterized by strong, positive loadings on acylcarnitines C18:1 and C18:2, amino acids glutamate and ornithine, and biogenic amine taurine. On treelet component 3, all eight lysophosphatidylcholines loaded strongly, positively. There was a positive correlation between treelet components 1 and 3 (*r* = 0.46), while treelet component 2 was not strongly correlated with the other patterns (Supporting Information Table S3).

### Treelet patterns and prostate cancer risk

**Figure 3:**
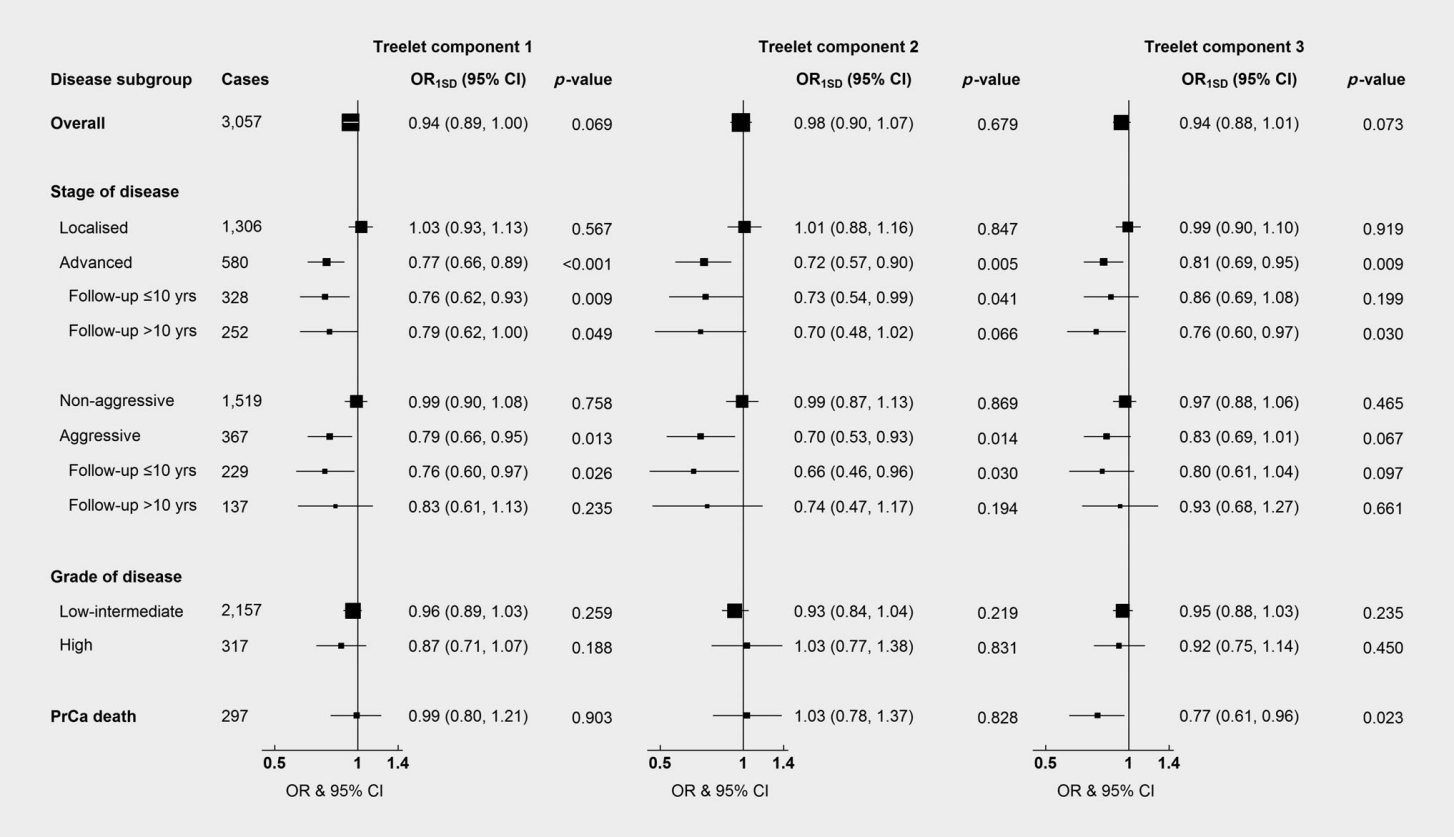
Odds ratio of prostate cancer overall and subgroups associated with a one standard deviation increase in treelet component scores. Stage and grade of prostate cancer were categorized using the tumor‐node‐metastasis (TNM) system and Gleason score, respectively; localized (≤T_2_ and N_0/x_ and M_0_, or stage coded as localized), advanced (T_3–4_ and/or N_1–3_ and/or M_1_, or coded as advanced), non‐aggressive (≤T_3_ and N_0/x_ and M_0_), aggressive (a subset of advanced stage disease defined as T_4_ and/or N_1–3_ and/or M_1_), low‐intermediate grade (Gleason <8 or coded as well, moderately or poorly differentiated tumors) and high grade (Gleason ≥8 or coded as undifferentiated tumors)_._ Death from prostate cancer during follow‐up was defined as prostate cancer listed as the underlying cause of death on the death certificate. One matched set was excluded from the analysis of aggressive prostate cancer with follow‐up >10 years; the case was the only individual with unknown smoking status in this subgroup and the model was thus not stable. Abbreviations: OR_1SD_, odds ratio for a one standard deviation increase in treelet component score; CI, confidence interval; yrs, years; PrCa, prostate cancer.

Associations of metabolite patterns and risk of prostate cancer are shown in Figure 3 and Supporting Information Table S4. No associations were observed for prostate cancer overall, nor with localized, non‐aggressive, low‐intermediate and high grade prostate cancer.

Treelet component 1 was inversely associated with risk of advanced stage prostate cancer (odds ratio for a SD increase in treelet component score [OR_1SD_] = 0.77, 95% confidence interval [CI] 0.66–0.89, *p* = 0.0007), suggesting that men with higher concentrations of diacyl and acyl‐alkyl phosphatidylcholines and three hydroxysphingomyelins had a lower risk of advanced stage prostate cancer (Fig. 3 and Supporting Information Table S4). The results were unchanged when stratifying at 10 years of follow‐up. Furthermore, treelet component 1 was associated with risk of aggressive prostate cancer (OR_1SD_ = 0.79, 95%CI 0.66–0.95, *p* = 0.013), with no heterogeneity by follow‐up time. In the sensitivity analysis further adjusting for treelet component 3, results for treelet component 1 remained largely unchanged, although the associations with advanced stage with more than 10 years of follow‐up and with aggressive prostate cancer were slightly attenuated and no longer statistically significant (Supporting Information Table S5).

Men who scored higher on treelet component 2, characterized by higher acylcarnitines C18:1 and C18:2, glutamate, ornithine and taurine, had a lower risk of advanced stage disease (OR_1SD_ = 0.72, 95%CI 0.57–0.90, *p* = 0.005; Figure 3 and Supporting Information Table S4). When stratifying by follow‐up time, risk estimates remained unchanged, but the significance was borderline for follow‐up beyond 10 years (OR_1SD_ = 0.70, 95%CI 0.48–1.02, *p* = 0.066), perhaps due to lower numbers. Moreover, this pattern was inversely associated with risk of aggressive prostate cancer (OR_1SD_ = 0.70, 95%CI 0.53–0.93, *p* = 0.014), with no heterogeneity by follow‐up time.

Treelet component 3, characterized by positive loadings on lysophosphatidylcholines, was inversely related to risk of advanced stage prostate cancer (OR_1SD_ = 0.81, 95%CI 0.69–0.95, *p* = 0.009; Figure 3 and Supporting Information Table S4). Risk estimates were similar when stratifying by time to diagnosis and remained significant for follow‐up beyond 10 years (OR_1SD_ = 0.76, 95%CI 0.60–0.97, *p* = 0.030). An inverse association with risk of death from prostate cancer was also observed (OR_1SD_ = 0.77, 95%CI 0.61–0.96, *p* = 0.023). When further adjusting for treelet component 1, the associations of treelet component 3 with advanced prostate cancer were slightly attenuated and no longer statistically significant, while the association with prostate cancer death remained (Supporting Information Table S5).

When restricting the analysis to 2,018 cases and their matched controls who were not included in our previous publication,^6^ we obtained similar results to those observed for the full study population (*n*_case_ = 3,057; Supporting Information Table S6).

### Principal component analysis

Treelet components 1 and 2, were similar to principal components 1 (*r* = 0.99) and 2 (*r* = 0.88), respectively (Supporting Information Tables S7 and S8). Treelet component 3 (lysophosphatidylcholines) was correlated with principal components 1 (*r* = 0.54), 5 (*r* = 0.43) and 9 (*r* = 0.38) but qualitatively most similar to principal component 9, which was mostly characterized by positive loadings on the lysophosphatidylcholines, some acylcarnitines (especially C14:1), amino acids arginine and t4‐hydroxyproline, and sphingomyelin SM C20:2, while having negative loadings on amino acid glutamate and several phosphatidylcholines.

The associations of principal components 1 and 2 with prostate cancer risk (Supporting Information Table S9) were similar to those observed for treelet components 1 and 2 (Fig. 3), respectively. Unlike treelet component 3, principal component 9 was not associated with advanced stage prostate cancer, but in line with the main results, a trend toward an inverse association with risk of prostate cancer death was observed (OR_1SD_ = 0.85, 95%CI 0.0.70–1.03, *p* = 0.095). Principal component 1 was inversely associated with risk of advanced (overall and by follow‐up time) and aggressive prostate cancer (with no heterogeneity by length of follow‐up), but not prostate cancer death, while principal component 5 was not related to risk of any prostate cancer outcome.

In addition, principal component 4, characterized by positive loadings on several acylcarnitines, amino acid t4‐hydroxyproline, biogenic amine sarcosine and several phosphatidylcholines, and inverse loadings on other phosphatidylcholines and sphingolipids, was inversely associated with advanced (with no heterogeneity by follow‐up time), aggressive (with a somewhat stronger association in men who had been followed up for more than 10 years) and high grade prostate cancer. Finally, principal component 8, mainly characterized by positive loadings on some acylcarnitines, arginine, t4‐hydroxyproline and SM C20:2, mixed loadings on the glycerophospholipids, and inverse loadings on all the hydroxysphingomyelins, was inversely associated with all prostate cancer outcomes, except advanced prostate cancer at longer follow‐up and prostate cancer death.

### Analysis of individual metabolites

Results of the associations between individual metabolite concentrations and prostate cancer risk in the full dataset (*n*_case_ = 3,057) and in the data not previously published^6^ (*n*_case_ = 2,018) are shown in Supporting Information Tables S10–S23. Results from the new dataset were in line with those of the full dataset, although slightly weaker, with two obvious exceptions; acyl‐alkyl phosphatidylcholines were more often associated with overall prostate cancer in the new dataset than in the full dataset (Supporting Information Tables S10 and S17), and the same was the case for diacyl phosphatidylcholines and risk of low‐intermediate grade prostate cancer (Supporting Information Tables S15 and S22).

## Discussion

In this large prospective study, treelet transform identified three easily interpretable patterns in men's metabolite profile. We observed a lower risk of advanced stage prostate cancer among men with metabolite profiles akin to those described by treelet components 1 and 2, characterised by higher concentrations of phosphatidylcholines and hydroxysphingomyelins (treelet component 1), or the combination of acylcarnitines C18:1 and C18:2, glutamate, ornithine and taurine (treelet component 2). Importantly for our understanding of potential etiological factors for prostate cancer, the risk estimates remained similar in case‐sets in which the case was diagnosed more than 10 years after blood collection. These two metabolite patterns showed similar associations with aggressive prostate cancer (the more aggressive subset of advanced stage prostate cancer), also with no heterogeneity by follow‐up time. Moreover, similar results were observed for men with higher concentrations of lysophosphatidylcholines (treelet component 3) but these associations were partly explained by confounding from the phosphatidylcholine and hydroxysphingomyelin pattern (treelet component 1), while the association of treelet component 3 with prostate cancer death may be independent of treelet component 1.

To the best of our knowledge, only one other prospective study of metabolites and prostate cancer risk has applied dimension‐reduction methods to the full set of metabolites. None of 10 metabolite patterns derived using PCA were related to risk of overall or aggressive prostate cancer (defined by combining stage and grade information) in observational analyses within the Prostate, Lung, Colorectal and Ovarian Cancer Screening Trial; 695 metabolites measured using mass spectrometry were included, but the metabolites which characterized each pattern were not described.^19^ Use of different metabolomics platforms, dimension‐reduction methods, study populations and tumor subtype categorization may partly explain the differences in results.

The clustering of phosphatidylcholine and sphingomyelins (treelet component 1) as well as the positive correlation between treelet components 1 and 3 (lysophosphatidylcholines) observed here may be explained by shared metabolic pathways; sphingomyelins are synthesized from phosphatidylcholines,^20^ and lysophosphatidylcholines are a subtype of phosphatidylcholines with one fatty acid side chain. In line with our results, treelet transform applied to untargeted metabolomics and lipidomics data from EPIC‐Potsdam also identified a pattern characterized by positive loadings on a sphingomyelin, three phosphatidylcholines and other glycerophospholipids^12^ (a class including phosphatidylcholines^21^). Diet, fitness level, alcohol intake and body mass index might be modifiable determinants of these metabolites.^22^, ^23^, ^24^, ^25^, ^26^

The current results on phosphatidylcholines and sphingomyelins (treelet component 1) and risk are broadly consistent with those of other prospective studies investigating individual metabolites, including our previously published results,^6^ which were conducted in a subset (~35%) of the current study population. For example, we previously reported inverse associations of many individual phosphatidylcholines, all of which were included with positive loadings in treelet component 1 or 3, with risk of advanced stage prostate cancer, and some associations remained when restricting the analysis to aggressive prostate cancer. We observed generally similar but less strong associations when restricting the analysis to just the new data included in the current analysis. Comparison with results from other studies is complex because of the use of different analytical platforms, for which only a limited number of metabolites (acylcarnitines, amino acids and biogenic amines) perfectly overlap with the metabolites studied here, and of a different classification of prostate cancer subtypes (aggressive prostate cancer was defined by combining information on stage and grade, in previous publications from other groups). Nonetheless, observational analyses within the Alpha‐Tocopherol, Beta‐Carotene Cancer Prevention Study (ATBC) found that men with higher concentrations of some glycerophospholipids had lower risk of aggressive prostate cancer^8^ and possibly tumor stage T3^27^; these results appear similar to our findings. In contrast, little is known about the association of lysophosphatidylcholines (treelet component 3) with risk of dying from prostate cancer. Another study in ATBC reported an inverse association between lysolipid 1‐linoleoyl‐glycerophosphatidylcholine (18:2), which is comparable to lysoPC a C18:2 included in TC3 of the current study, and risk of death from prostate cancer (*n*_deaths_ = 523),^10^ but death was not an outcome in the other previous papers.

The metabolite classes included in treelet components 1 and 3 are structural components of cell membranes, and along with the related enzymes and intermediates they are involved in proliferation, cell signaling and cell survival, and thus may play key roles in carcinogenesis, progression and migration of tumors.^20^, ^21^, ^28^ Their potential association may apply to cancer in general rather than being prostate cancer‐specific; some prospective studies have reported lower risk of breast cancer,^9^, ^29^ colorectal cancer,^30^ hepatocellular carcinoma^31^, ^32^ and pancreatic cancer^33^ in relation to higher levels of phosphatidylcholines and related metabolites, while another study did not observe an association between the few included phospholipids and breast cancer risk.^34^

Involvement in shared metabolic pathways may also explain the grouping of metabolites in treelet component 2. Acylcarnitines are involved in β‐oxidation of fatty acids, which produces acetyl coenzyme A for energy production via the Krebs cycle.^35^ Glutamate also feeds into this cycle by conversion to α‐ketoglutarate but can also be converted to ornithine. Moreover, taurine has been proposed to stimulate β‐oxidation and support mitochondrial function.^36^ Thus, alteration in energy metabolism could be one possible underlying mechanism of the observed associations between treelet component 2 and reduced risk of advanced stage and aggressive prostate cancer. In line with our results, analyses in ATBC showed an inverse association between metabolites related to energy metabolism (α‐ketoglutarate and citrate) and risk of aggressive prostate cancer.^7^, ^8^ Our previous study in EPIC also suggested inverse associations of acylcarnitines and taurine with advanced prostate cancer.^6^ There are also some published data on dietary and lifestyle correlates of the metabolites in treelet component 2.^23^, ^26^, ^37^, ^38^

Altered levels of phosphatidylcholines, lysophospholipids (mostly lower), sphingomyelins, acylcarnitines, glutamate, ornithine (mostly lower) and taurine in postdiagnostic samples from prostate cancer patients have been reported.^4^, ^39^, ^40^ The most recent of these studies, used Mendelian randomization to assess the causality of cross‐sectionally observed associations between 14 serum metabolites (including tyrosine), measured using nuclear magnetic resonance spectroscopy, and overall risk of prostate cancer, detected via prostate‐specific antigen test.^40^ No associations between genetic markers of these metabolites (explaining a median of 6.8% [range 0.4–11.2%] of the variance in metabolite concentrations^41^) and risk of prostate cancer were observed.^40^ This suggests no evidence of causality in the associations between these specific metabolites and risk of prostate cancer overall. While these case–control studies could imply that our results may be driven by tumors already present at blood collection, our prospective analysis excluding the first 10 years of follow‐up suggests that differences in metabolite concentrations may precede carcinogenesis and thus potentially be relevant for the etiology of prostate cancer.

This study benefits from the large, mature and well‐described cohort, which provided more statistical power than previous papers, and limited potential reverse causation and confounding. Additionally, treelet transform was applied to reduce issues of multiple testing and correlated variables pertaining to univariate analyses, while producing interpretable and stable metabolite patterns. For comparison, we also derive metabolite patterns using the more common PCA, and associations of the first and second principal components with prostate cancer risk were similar to those of the first and second treelet components, respectively. The advantage of treelet transform for analysis of metabolomics data in epidemiological studies has further been highlighted by a study also observing that treelet components were similar to and easier to interpret than components from PCA, albeit with somewhat less variance explained.^12^ A limitation of treelet transform is the need to select a cut‐level to produce components. However, the choice is aided by data‐driven cross‐validation, and the sensitivity analysis of the cut‐level produced similar components.

Further limitations of this study are related to the blood sampling, handing and assay procedure. Only one blood sample was available per participant, which may lead to attenuation of risk estimates if a single measurement does not represent long‐term exposure. Although studies of short‐term reproducibility suggest that one measurement may be adequate for most metabolites (median intercorrelation coefficients of 0.45–0.70 over 0.3–2.3 years),^42^, ^43^, ^44^, ^45^ long‐term reproducibility is lower (mean *r* = 0.28, 0.13 and 0.16 over 15 years for all measured metabolites, diacyl and acyl‐alkyl phosphatidylcholines, respectively).^44^ Moreover, differences in sample handling procedures between study centers and the use of nonfasting blood samples might potentially have attenuated our risk estimates. To minimize this, cases and controls were matched on study center and fasting status, and the latter has been shown to explain only a small amount of variability in metabolite concentrations.^23^, ^46^, ^47^, ^48^

In conclusion, in this the largest study to date, we found evidence that easily interpretable patterns in baseline plasma metabolite profile, identified using treelet transform, are associated with subsequent risk of more aggressive prostate cancer subtypes. Men with metabolite profiles characterized by higher concentrations of either phosphatidylcholines and hydroxysphingomyelins, or acylcarnitines C18:1 and C18:2, glutamate, ornithine and taurine, had lower risk of advanced and aggressive stage prostate cancer. Differences in scores on these metabolite patterns between cases and controls may precede diagnosis of advanced stage prostate cancer by more than 10 years and thus might be relevant to further understanding of prostate cancer etiology and prevention. Moreover, men with metabolite profiles high in lysophosphatidylcholines may be at lower risk of dying from prostate cancer.

## Availability of data and materials

For information on how to submit an application for gaining access to EPIC data and/or biospecimens, please follow the instructions at http://epic.iarc.fr/access/index.php

## Disclaimer

Where authors are identified as personnel of the International Agency for Research on Cancer/World Health Organization, the authors alone are responsible for the views expressed in this article and they do not necessarily represent the decisions, policy or views of the International Agency for Research on Cancer/World Health Organization.

## Supporting information


**Appendix S1:** Supporting Information.

**Figure S1** Scree plot for the final treelet transform. Concentrations of 119 metabolites for 3,057 control participants from EPIC were included. Three treelet components were retained and cut‐level 97 was used.

**Table S1** Coefficients of variation for metabolites
**Table S2**. Loadings for the original metabolites on three treelet components derived using treelet transform at cut‐level 97 in 3,057 control participants from EPIC
**Table S3**. Correlations between treelet component scores in 3,057 control participants in EPIC
**Table S4**. Risk of prostate cancer in relation to treelet component scores in 3,057 matched case–control sets from EPIC
**Table S5**. Risk of prostate cancer in relation to treelet component scores for TC1 and TC3 mutually adjusted, in 3,057 matched case–control sets from EPIC
**Table S6**. Risk of overall prostate cancer in relation to treelet component scores, in 2018 matched case–control sets from EPIC
**Table S7**. Loadings for the original metabolites on nine principal components derived using principal component analysis in 3,057 control participants from EPIC
**Table S8**. Correlations between scores for metabolite patterns derived using treelet transform and principal component analysis in 3,057 control participants from EPIC
**Table S9**. Risk of prostate cancer in relation to principal component scores in 3,057 matched case–control sets from EPIC

**Table S10** Full dataset: Risk of overall prostate cancer per 1 SD increase in metabolite concentrations in EPIC^1^, *n*_case_ = 3,057
**Table S11**. Full dataset: Risk of prostate cancer by stage per 1 SD increase in metabolite concentrations in EPIC^1^
**Table S12**. Full dataset: Risk of advanced prostate cancer by follow‐up time per 1 SD increase in metabolite concentrations in EPIC^1^
**Table S13**. Full dataset: Risk of prostate cancer by aggressiveness per 1 SD increase in metabolite concentrations in EPIC^1^
**Table S14**. Full dataset: Risk of aggressive prostate cancer by follow‐up time per 1 SD increase in metabolite concentrations in EPIC^1^
**Table S15**. Full dataset: Risk of prostate cancer by grade per 1 SD increase in metabolite concentrations in EPIC^1^
**Table S16**. Full dataset: Risk of prostate cancer death per 1 SD increase in metabolite concentrations in EPIC^1^, *n*_case_ = 297^2^
**Table S17**. New dataset: Risk of overall prostate cancer per 1 SD increase in metabolite concentrations in EPIC^1^, *n*_case_ = 2018
**Table S18**. New dataset: Risk of prostate cancer by stage per 1 SD increase in metabolite concentrations in EPIC^1^
**Table S19**. New dataset: Risk of advanced prostate cancer by follow‐up time per 1 SD increase in metabolite concentrations in EPIC^1^
**Table S20**. New dataset: Risk of prostate cancer by aggressiveness per 1 SD increase in metabolite concentrations in EPIC^1^
**Table S21**. New dataset: Risk of aggressive prostate cancer by follow‐up time per 1 SD increase in metabolite concentrations in EPIC^1^
**Table S22**. New dataset: Risk of prostate cancer by grade per 1 SD increase in metabolite concentrations in EPIC^1^
**Table S23**. New dataset: Risk of prostate cancer death per 1 SD increase in metabolite concentrations in EPIC^1^, *n*_case_ = 157^2^
