# Supplementary material for: Patterns in metabolite profile are associated with risk of more aggressive prostate cancer: A prospective study of 3,057 matched case–control sets from EPIC
Source: Int J Cancer. 2019 Apr 29;146(3):720–30. doi: 10.1002/ijc.32314 (PMC6916595; doi:10.1002/ijc.32314)
Supplement: Supplementary file 1 — Appendix S1: Supporting Information. [file IJC-146-720-s001.doc]

# Supporting information - methods

Contents

[Exclusions of metabolites and men for the current analysis 1](#__RefHeading___Toc536107366)

[Supporting Information – Methods, Table 1 2](#__RefHeading___Toc536107367)

[Supporting Information – Methods, Table 2 2](#__RefHeading___Toc536107368)

[Metabolite nomenclature 2](#__RefHeading___Toc536107369)

[Statistical analysis 3](#__RefHeading___Toc536107370)

[Sensitivity of cut-level in treelet transform 3](#__RefHeading___Toc536107371)

[Stability assessment of treelet components 3](#__RefHeading___Toc536107372)

[Supporting Information – Methods, Table 3 4](#__RefHeading___Toc536107373)

[Quintiles of treelet component scores 8](#__RefHeading___Toc536107377)

[Test for heterogeneity in the association between treelet components and prostate cancer risk 8](#__RefHeading___Toc536107378)

[Principal component analysis 8](#__RefHeading___Toc536107379)

[Analysis of individual metabolite concentrations 9](#__RefHeading___Toc536107380)

[References..............................................................................................................................................9](#__RefHeading___Toc536107381)

## Exclusions of metabolites and men for the current analysis

Exclusions of metabolites were made separately for each of five assay rounds before data were combined. Depending on assay round, 142 to 146 metabolite were quantified (Supporting Information – Methods, Table 1), which was a total of 148 metabolites across the assay rounds. Metabolites were excluded if more than 20% of men had non-quantifiable assay results (missing data or results outside the measurable range; n=14 to 17 metabolites) or if the overall coefficient of variation (across all blinded quality control samples within an assay round) was higher than 20% (n=0 to 5 metabolites); coefficients of variation are seen in Table S1. Of the remaining metabolites (n=123 to 130), 119 were available from all assay rounds and thus included in the statistical analyses.

**Supporting Information – Methods, Table 1. Exclusion of metabolites**

| **Assay round** | **Metabolite returned from lab, n** | **Exclusions** | | **Metabolites left, n** | **Overlap between assay rounds, and thus left for statistical analysis, n** |
| --- | --- | --- | --- | --- | --- |
|  |  | **>20% non-quantifiable measurements, n** | **Coefficient of variation > 20%, n** |  |  |
| 1 | 142 | 17 | 2 | 123 | 119 |
| 2 | 144 | 15 | 2 | 127 |  |
| 3 | 146 | 17 | 5 | 124 |  |
| 4 | 143 | 14 | 0 | 129 |  |
| 5 | 144 | 14 | 0 | 130 |  |

Metabolite data were available for a total of 6132 men. Men with missing information on any of the 119 metabolites (n=1 man) and men in incomplete case-control sets (n=17 men) were excluded, leaving 3057 matched case-control sets in the statistical analyses (Supporting Information – Methods, Table 2).

Supporting Information – Methods, Table 2**. Exclusions of participants**

|  | **Exclusions, n** | **Men left, n** |
| --- | --- | --- |
| **Samples sent to the laboratory** |  | **6137** |
| Insufficient sample | 4 |  |
| Analytic issue | 1 |  |
| **Men with metabolite data** |  | **6132** |
| Missing on included metabolites | 1 |  |
| Not in matched sets | 17 |  |
| **Men in the statistical analysis** |  | **6114** |

##

## Metabolite nomenclature

Fatty acid side chains in acylcarnitines, glycerophospholipids and sphingolipids were labelled ‘Cx:y’, where ‘x’ and ‘y’ denote the total number of carbon atoms and double bonds, respectively, in each molecule [1]. Acylcarnitines were abbreviated according to the fatty acid side chain. All glycerophospholipids were phosphatidylcholines, and sub-classes were separated by the number and type of fatty acids side chains. ‘LysoPC a’ denotes phosphatidylcholines with one acyl fatty acid side chain, ‘PC aa’ denotes two acyl side chains (diacyls), and ‘PC ae’ denotes one acyl and one alkyl side chain (acyl-alkyl). Sphingolipids were all sphingomyelins (SM) with or without a hydroxy group (OH) attached and were also labelled according to the fatty acid side chain. Hexose is the sum of a range of monosaccharides with six carbon atoms, including glucose, fructose and galactose.

## Statistical analysis

## *Sensitivity of cut-level in treelet transform*

A sensitivity analysis of the cut-level was performed by identifying treelet components at ±3 cut-levels [2]. Cutting the cluster tree at 94 compared to 97 resulted in three fewer diacylphosphatidylcholines (PC aa C42:0, PC aa C42:1 and PC aa C42:4) and eleven fewer acyl-alkylphosphatidylcholines (PC ae C36:4, PC ae C36:5, PC ae C38:4, PC ae C38:5, PC ae C40:4, PC ae C40:5, PC ae C42:4, PC ae C42:5, PC ae C44:4, PC ae C44:5 and PC ae C44:6) being included in treelet component 1 (TC1), while TC2 and TC3 were identical to those of the main treelet transform. Using cut-level 100 produced three TCs identical to the ones created at cut-level 97.

## *Stability assessment of treelet components*

The stability of components was assessed by examining the sign patterns of component loadings in 100 repeat random sub-samples, comprising 80% of the control data (n=2445) [3]. The sign pattern is determined by the signs of the loadings (e.g. the sign pattern for a component with loadings ‘0.1, 0.0, 0.3, …, -0,2’ is ‘+, 0, +, …, -’). The more frequent a sign pattern is, the more stable is the corresponding component.

The stability assessment of the treelet components, suggested high stability because the sign patterns had high frequencies and were very similar to the treelet components (Supporting Information – Methods, Table 3). The frequencies of sign patterns 1 and 4, which mirrored TC1 and TC3 respectively, were as high as possible (1.00). The frequency of sign pattern 2, which matched TC2, was lower (0.58) but in combination with sign pattern 3, which only included one additional acylcarnitine (C16:0), the frequency was also 1.00. Moreover, the ranking of all the sign patterns mirrored that of the final TCs.

Supporting Information – Methods, Table 3**. Stability assessment of three treelet components at cut-level 97 in 3057 control participants in EPIC.**

**A. Rank, frequency, variance and comparison of sign patterns with the treelet components.**

| **Sign pattern** | **Average rank** | **Frequency** | **Average variance** | **Fit with treelet component** |
| --- | --- | --- | --- | --- |
| 1 | 1.00 | 1.00 | 2.405 | Perfect match with TC1 |
| 2 | 2.00 | 0.58 | 0.581 | Perfect match with TC2 |
| 3 | 2.00 | 0.42 | 0.597 | Includes all metabolites in TC2 and acylcarnitine C16 |
| 4 | 3.00 | 1.00 | 0.524 | Perfect match with TC3 |

Abbreviations: EPIC, European Prospective Investigation into Cancer and Nutrition; TC, treelet component

**B. The sign patterns of the loadings of each original metabolite.**

| **Metabolites** | **Sign patterns** | | | |
| --- | --- | --- | --- | --- |
|  | **1** | **2** | **3** | **4** |
| **ACYLCARNITINES** |  |  |  |  |
| C0 | 0 | 0 | 0 | 0 |
| C2 | 0 | 0 | 0 | 0 |
| C3 | 0 | 0 | 0 | 0 |
| C14:1 | 0 | 0 | 0 | 0 |
| C16 | 0 | 0 | + | 0 |
| C18 | 0 | 0 | 0 | 0 |
| C18:1 | 0 | + | + | 0 |
| C18:2 | 0 | + | + | 0 |
| **AMINO ACIDS** |  |  |  |  |
| Alanine | 0 | 0 | 0 | 0 |
| Arginine | 0 | 0 | 0 | 0 |
| Asparagine | 0 | 0 | 0 | 0 |
| Citrulline | 0 | 0 | 0 | 0 |
| Glutamate | 0 | + | + | 0 |
| Glutamine | 0 | 0 | 0 | 0 |
| Glycine | 0 | 0 | 0 | 0 |
| Histidine | 0 | 0 | 0 | 0 |
| Isoleucine | 0 | 0 | 0 | 0 |
| Leucine | 0 | 0 | 0 | 0 |
| Lysine | 0 | 0 | 0 | 0 |
| Methionine | 0 | 0 | 0 | 0 |
| Ornithine | 0 | + | + | 0 |
| Phenylalanine | 0 | 0 | 0 | 0 |
| Proline | 0 | 0 | 0 | 0 |
| Serine | 0 | 0 | 0 | 0 |
| t4-hydroxyproline | 0 | 0 | 0 | 0 |
| Threonine | 0 | 0 | 0 | 0 |
| Tryptophan | 0 | 0 | 0 | 0 |
| Tyrosine | 0 | 0 | 0 | 0 |
| Valine | 0 | 0 | 0 | 0 |
| **BIOGENIC AMINES** | |  |  |  |
| ADMA | 0 | 0 | 0 | 0 |
| Creatinine | 0 | 0 | 0 | 0 |
| Kynurenine | 0 | 0 | 0 | 0 |
| Sarcosine | 0 | 0 | 0 | 0 |
| Taurine | 0 | + | + | 0 |
| **GLYCEROPHOSPHOLIPIDS** | |  |  |  |
| **Lysophosphatidylcholines** | |  |  |  |
| Lyso PC a C16:0 | 0 | 0 | 0 | + |
| Lyso PC a C16:1 | 0 | 0 | 0 | + |
| Continues | | | | |
| Supporting Information – Methods, Table 3**B continued** | | | | |
| **Metabolites** | **Sign patterns** | | | |
|  | **1** | **2** | **3** | **4** |
| Lyso PC a C17:0 | 0 | 0 | 0 | + |
| Lyso PC a C18:0 | 0 | 0 | 0 | + |
| Lyso PC a C18:1 | 0 | 0 | 0 | + |
| Lyso PC a C18:2 | 0 | 0 | 0 | + |
| Lyso PC a C20:3 | 0 | 0 | 0 | + |
| Lyso PC a C20:4 | 0 | 0 | 0 | + |
| **Diacyl-phosphatidylcholines** | |  |  |  |
| PC aa C28:1 | + | 0 | 0 | 0 |
| PC aa C30:0 | + | 0 | 0 | 0 |
| PC aa C32:0 | + | 0 | 0 | 0 |
| PC aa C32:1 | + | 0 | 0 | 0 |
| PC aa C32:3 | + | 0 | 0 | 0 |
| PC aa C34:1 | + | 0 | 0 | 0 |
| PC aa C34:2 | + | 0 | 0 | 0 |
| PC aa C34:3 | + | 0 | 0 | 0 |
| PC aa C34:4 | + | 0 | 0 | 0 |
| PC aa C36:0 | + | 0 | 0 | 0 |
| PC aa C36:1 | + | 0 | 0 | 0 |
| PC aa C36:2 | + | 0 | 0 | 0 |
| PC aa C36:3 | + | 0 | 0 | 0 |
| PC aa C36:4 | + | 0 | 0 | 0 |
| PC aa C36:5 | + | 0 | 0 | 0 |
| PC aa C36:6 | + | 0 | 0 | 0 |
| PC aa C38:0 | + | 0 | 0 | 0 |
| PC aa C38:3 | + | 0 | 0 | 0 |
| PC aa C38:4 | + | 0 | 0 | 0 |
| PC aa C38:5 | + | 0 | 0 | 0 |
| PC aa C38:6 | + | 0 | 0 | 0 |
| PC aa C40:2 | + | 0 | 0 | 0 |
| PC aa C40:3 | + | 0 | 0 | 0 |
| PC aa C40:4 | + | 0 | 0 | 0 |
| PC aa C40:5 | + | 0 | 0 | 0 |
| PC aa C40:6 | + | 0 | 0 | 0 |
| PC aa C42:0 | + | 0 | 0 | 0 |
| PC aa C42:1 | + | 0 | 0 | 0 |
| PC aa C42:2 | + | 0 | 0 | 0 |
| PC aa C42:4 | + | 0 | 0 | 0 |
| PC aa C42:5 | + | 0 | 0 | 0 |
| **Acyl-alkyl-phosphatidylcholines** | | |  |  |
| PC ae C30:0 | + | 0 | 0 | 0 |
| PC ae C30:2 | + | 0 | 0 | 0 |
| Continues |  |  |  |  |
| Supporting Information – Methods, Table 3**B continued** | | | | |
| **Metabolites** | **Sign patterns** | | | |
|  | **1** | **2** | **3** | **4** |
| PC ae C32:1 | + | 0 | 0 | 0 |
| PC ae C32:2 | + | 0 | 0 | 0 |
| PC ae C34:0 | + | 0 | 0 | 0 |
| PC ae C34:1 | + | 0 | 0 | 0 |
| PC ae C34:2 | + | 0 | 0 | 0 |
| PC ae C34:3 | + | 0 | 0 | 0 |
| PC ae C36:0 | + | 0 | 0 | 0 |
| PC ae C36:1 | + | 0 | 0 | 0 |
| PC ae C36:2 | + | 0 | 0 | 0 |
| PC ae C36:3 | + | 0 | 0 | 0 |
| PC ae C36:4 | + | 0 | 0 | 0 |
| PC ae C36:5 | + | 0 | 0 | 0 |
| PC ae C38:2 | + | 0 | 0 | 0 |
| PC ae C38:3 | + | 0 | 0 | 0 |
| PC ae C38:4 | + | 0 | 0 | 0 |
| PC ae C38:5 | + | 0 | 0 | 0 |
| PC ae C38:6 | + | 0 | 0 | 0 |
| PC ae C40:1 | + | 0 | 0 | 0 |
| PC ae C40:2 | + | 0 | 0 | 0 |
| PC ae C40:3 | + | 0 | 0 | 0 |
| PC ae C40:4 | + | 0 | 0 | 0 |
| PC ae C40:5 | + | 0 | 0 | 0 |
| PC ae C40:6 | + | 0 | 0 | 0 |
| PC ae C42:1 | + | 0 | 0 | 0 |
| PC ae C42:2 | + | 0 | 0 | 0 |
| PC ae C42:3 | + | 0 | 0 | 0 |
| PC ae C42:4 | + | 0 | 0 | 0 |
| PC ae C42:5 | + | 0 | 0 | 0 |
| PC ae C44:4 | + | 0 | 0 | 0 |
| PC ae C44:5 | + | 0 | 0 | 0 |
| PC ae C44:6 | + | 0 | 0 | 0 |
| **HEXOSE** |  |  |  |  |
| Hexose | 0 | 0 | 0 | 0 |
| **SPHINGOLIPIDS** |  |  |  |  |
| **Hydroxysphingomyelins** | |  |  |  |
| SM (OH) C14:1 | + | 0 | 0 | 0 |
| SM (OH) C16:1 | + | 0 | 0 | 0 |
| SM (OH) C22:1 | 0 | 0 | 0 | 0 |
| SM (OH) C22:2 | + | 0 | 0 | 0 |
| SM (OH) C24:1 | 0 | 0 | 0 | 0 |

Continues

| Supporting Information – Methods, Table 3**B continued** | | | | |
| --- | --- | --- | --- | --- |
| **Metabolites** | **Sign patterns** | | | |
|  | **1** | **2** | **3** | **4** |
| **Sphingomyelins** |  |  |  |  |
| SM C16:0 | 0 | 0 | 0 | 0 |
| SM C16:1 | 0 | 0 | 0 | 0 |
| SM C18:0 | 0 | 0 | 0 | 0 |
| SM C18:1 | 0 | 0 | 0 | 0 |
| SM C20:2 | 0 | 0 | 0 | 0 |
| SM C24:0 | 0 | 0 | 0 | 0 |
| SM C24:1 | 0 | 0 | 0 | 0 |

^1^ One hundred repeat treelet transforms were performed in 80% sub-samples of the control study population (n=2445).

## *Quintiles of treelet component scores*

The categorisation of treelet component scores into quintiles was based on the overall distribution among controls. Using assay-round specific quartiles in controls did not materially change the results, and thus overall quartiles were used in all analyses.

## *Test for heterogeneity in the association between treelet components and prostate cancer risk*

Tests for heterogeneity in the associations between treelet components and prostate cancer risk by subgroups according to stage and grade were done using the likelihood ratio χ^2^ test, which compared models with and without an interaction term between the linear trend variables and the outcome variable of interest, e.g. grade.

## *Principal component analysis*

For comparison with the metabolite patterns derived using treelet transform, we conducted an analysis using the more commonly used method, principal component analysis (PCA) to derive metabolite patterns. As for the treelet transform, log-transformed metabolite concentrations were entered in the PCA and the analysis was based on the covariance matrix. The first nine principal components were retained because principal components 1, 5 and 9 had some similarity with one or more of the three treelet components in the main analysis (Tables S7 and S8).

Pearson correlations coefficients were computed between each of the three treelet component and the nine principal components.

The retained principal components were entered in the conditional logistic regression model as described for the treelet components.

## *Analysis of individual metabolite concentrations*

In addition to the main analysis of metabolite patterns and risk of prostate cancer, results are presented for the individual association between each of the original 119 metabolite concentrations and risk of prostate cancer, in order to facilitate comparison with previous studies. These analyses were conducted using the same conditional logistic regression models as described for the main analyses.

Correction for multiple testing was applied using the Benjamini-Hochberg false discovery rate controlling procedure [4] with 0.05 as the significance level (α), which allows 5% of positive findings to be false, on average [5].

## References

1. Floegel A, Drogan D, Wang-Sattler R, Prehn C, Illig T, Adamski J, Joost HG, Boeing H, Pischon T. Reliability of serum metabolite concentrations over a 4-month period using a targeted metabolomic approach. PLoS One 2011; 6: e21103

2. Gorst-Rasmussen A, Dahm CC, Dethlefsen C, Scheike T, Overvad K. Exploring dietary patterns by using the treelet transform. Am J Epidemiol 2011; 173: 1097-1104

3. Gorst-Rasmussen A. tt: Treelet transform with Stata. Stata Journal 2012; 12: 130-146

4. Benjamini Y, Hochberg Y. Controlling the False Discovery Rate: A Practical and Powerful Approach to Multiple Testing. J R Stat Soc Series B Methodol 1995; 57: 289-300

5. Chadeau-Hyam M, Campanella G, Jombart T, Bottolo L, Portengen L, Vineis P, Liquet B, Vermeulen RC. Deciphering the complex: methodological overview of statistical models to derive OMICS-based biomarkers. Environ Mol Mutagen 2013; 54: 542-557
