# Supplementary material for: Patterns in metabolite profile are associated with risk of more aggressive prostate cancer: A prospective study of 3,057 matched case–control sets from EPIC
Source: Int J Cancer. 2019 Apr 29;146(3):720–30. doi: 10.1002/ijc.32314 (PMC6916595; doi:10.1002/ijc.32314)
Supplement: Supplementary file 2 — Figure S1 Scree plot for the final treelet transform. Concentrations of 119 metabolites for 3,057 control participants from EPIC were included. Three treelet components were retained and cut‐level 97 was used. [file IJC-146-720-s002.doc]

# Supporting Information – Figure S1

**Figure S1. Scree plot for the final treelet transform.**Concentrations of 119 metabolites for 3057 control participants from EPIC were included. Three treelet components were retained and cut-level 97 was used.
