# Supplementary material for: Patterns in metabolite profile are associated with risk of more aggressive prostate cancer: A prospective study of 3,057 matched case–control sets from EPIC
Source: Int J Cancer. 2019 Apr 29;146(3):720–30. doi: 10.1002/ijc.32314 (PMC6916595; doi:10.1002/ijc.32314)
Supplement: Supplementary file 3 — Table S1 Coefficients of variation for metabolites Table S2. Loadings for the original metabolites on three treelet components derived using treelet transform at cut‐level 97 in 3,057 control participants from EPIC Table S3. Correlations between treelet component scores in 3,057 control participants in EPIC Table S4. Risk of prostate cancer in relation to treelet component scores in 3,057 matched case–control sets from EPIC Table S5. Risk of prostate cancer in relation to treelet component scores for TC1 and TC3 mutually adjusted, in 3,057 matched case–control sets from EPIC Table S6. Risk of overall prostate cancer in relation to treelet component scores, in 2018 matched case–control sets from EPIC Table S7. Loadings for the original metabolites on nine principal components derived using principal component analysis in 3,057 control participants from EPIC Table S8. Correlations between scores for metabolite patterns derived using treelet transform and principal component analysis in 3,057 control participants from EPIC Table S9. Risk of prostate cancer in relation to principal component scores in 3,057 matched case–control sets from EPIC [file IJC-146-720-s003.doc]

# Supporting information – Tables S1-S9

Contents

[Table S1. Coefficients of variation for metabolites 2](#__RefHeading___Toc2256757)

[Table S2. Loadings for the original metabolites on three treelet components derived using treelet transform at cut-level 97 in 3057 control participants from EPIC 6](#__RefHeading___Toc2256758)

[Table S3. Correlations between treelet component scores in 3057 control participants in EPIC 8](#__RefHeading___Toc2256759)

[Table S4. Risk of prostate cancer in relation to treelet component scores in 3057 matched case-control sets from EPIC 9](#__RefHeading___Toc2256760)

[Table S5. Risk of prostate cancer in relation to treelet component scores for TC1 and TC3 mutually adjusted, in 3057 matched case-control sets from EPIC 11](#__RefHeading___Toc2256761)

[Table S6. Risk of overall prostate cancer in relation to treelet component scores, in 2018 matched case-control sets from EPIC 12](#__RefHeading___Toc2256762)

[Table S7. Loadings for the original metabolites on nine principal components derived using principal component analysis in 3057 control participants from EPIC 13](#__RefHeading___Toc2256763)

[Table S8. Correlations between scores for metabolite patterns derived using treelet transform and principal component analysis in 3057 control participants from EPIC 15](#__RefHeading___Toc2256764)

[Table S9. Risk of prostate cancer in relation to principal component scores in 3057 matched case-control sets from EPIC 16](#__RefHeading___Toc2256765)

[Reference 19](#__RefHeading___Toc2256766)

## Table S1. Coefficients of variation for metabolites

| **Metabolites** | **CV^1^** | | **n_QC_^2^** |
| --- | --- | --- | --- |
|  | **Min** | **Max** |  |
| **ACYLCARNITINES** |  |  |  |
| C0 | 4.89 | 7.96 | 568 |
| C2 | 4.99 | 9.26 | 560 |
| C3 | 5.26 | 10.29 | 560 |
| C3-DC (C4-OH) | 13.86 | 13.86 | 248 |
| C4 | 4.76 | 13.41 | 568 |
| C4:1 | 13.15 | 13.15 | 248 |
| C5 | 5.83 | 9.78 | 568 |
| C5-DC (C6-OH) | 16.84 | 16.84 | 8 |
| C5-M-DC | - | - | - |
| C8 | - | - | - |
| C10 | 8.24 | 8.24 | 8 |
| C10:1 | 6.22 | 6.98 | 416 |
| C12 | 8.74 | 10.01 | 130 |
| C12:1 | 9.88 | 13.02 | 468 |
| C14 | 0.00 | 5.60 | 60 |
| C14:1 | 6.02 | 16.65 | 568 |
| C14:2 | 3.01 | 14.15 | 476 |
| C16 | 5.07 | 13.83 | 568 |
| C16:1 | 5.93 | 5.93 | 8 |
| C18 | 5.66 | 15.81 | 568 |
| C18:1 | 6.13 | 11.69 | 560 |
| C18:2 | 6.92 | 17.19 | 568 |
| **AMINO ACIDS** |  |  |  |
| Alanine | 5.66 | 7.45 | 568 |
| Arginine | 7.24 | 9.66 | 568 |
| Asparagine | 5.39 | 10.23 | 568 |
| Aspartate | 13.41 | 18.69 | 476 |
| Citrulline | 6.17 | 13.65 | 568 |
| Glutamate | 7.64 | 11.88 | 568 |
| Glutamine | 7.06 | 13.10 | 568 |
| Glycine | 8.18 | 12.42 | 568 |
| Histidine | 6.71 | 8.34 | 568 |
| Isoleucine | 6.49 | 12.91 | 568 |
| Leucine | 6.53 | 9.90 | 568 |
| Lysine^3^ | 11.93 | 22.63 | 568 |
| Methionine | 10.06 | 19.55 | 568 |
| Ornithine | 11.71 | 18.33 | 568 |
| Phenylalanine | 6.31 | 10.01 | 568 |
| Proline | 6.03 | 8.91 | 568 |
| Serine | 7.61 | 8.69 | 568 |
| t4-hydroxyproline | 7.13 | 11.43 | 568 |
| Continues |  |  |  |
| **Table S1 continued** |  |  |  |
| **Metabolites** | **CV^1^** | | **n_QC_^2^** |
|  | **Minimum** | **Maximum** |  |
| Threonine | 5.95 | 8.50 | 568 |
| Tryptophan | 6.25 | 9.94 | 568 |
| Tyrosine | 6.26 | 9.82 | 568 |
| Valine | 10.90 | 12.93 | 568 |
| **BIOGENIC AMINES** |  |  |  |
| ADMA | 12.81 | 17.29 | 568 |
| alpha-AAA | 2.28 | 22.57 | 416 |
| Creatinine | 2.19 | 5.70 | 568 |
| Kynurenine | 10.78 | 13.03 | 568 |
| Putrescine | - | - | - |
| Sarcosine | 5.93 | 12.33 | 568 |
| SDMA | 8.83 | 20.52 | 568 |
| Serotonin | 4.98 | 4.98 | 52 |
| Spermidine | - | - | - |
| Spermine | - | - | - |
| Taurine | 4.30 | 7.24 | 568 |
| **GLYCEROPHOSPHOLIPIDS** |  |  |  |
| **Lysophosphatidylcholines** |  |  |  |
| Lyso PC a C16:0 | 5.58 | 10.45 | 568 |
| Lyso PC a C16:1 | 4.40 | 9.88 | 568 |
| Lyso PC a C17:0 | 6.07 | 10.16 | 568 |
| Lyso PC a C18:0 | 6.02 | 10.27 | 568 |
| Lyso PC a C18:1 | 5.31 | 9.90 | 568 |
| Lyso PC a C18:2 | 4.18 | 9.55 | 568 |
| Lyso PC a C20:3 | 6.98 | 13.60 | 568 |
| Lyso PC a C20:4 | 4.90 | 12.03 | 568 |
| lysoPC a C28:1 | 8.32 | 26.20 | 568 |
| **Diacylphosphatidylcholines** |  |  |  |
| PC aa C28:1 | 5.00 | 10.05 | 568 |
| PC aa C30:0 | 6.07 | 9.63 | 568 |
| PC aa C32:0 | 6.43 | 11.39 | 568 |
| PC aa C32:1 | 6.13 | 13.08 | 568 |
| PC aa C32:2 | 6.66 | 22.40 | 568 |
| PC aa C32:3 | 6.12 | 13.87 | 568 |
| PC aa C34:1 | 5.95 | 11.52 | 568 |
| PC aa C34:2 | 6.21 | 12.37 | 568 |
| PC aa C34:3 | 5.80 | 11.43 | 568 |
| PC aa C34:4 | 5.61 | 14.16 | 568 |
| PC aa C36:0 | 7.03 | 18.56 | 568 |
| PC aa C36:1 | 6.09 | 13.11 | 568 |
| PC aa C36:2 | 5.74 | 11.77 | 568 |
| PC aa C36:3 | 6.32 | 11.92 | 568 |
| Continues |  |  |  |
| **Table S1 continued** |  |  |  |
| **Metabolites** | **CV^1^** | | **n_QC_^2^** |
|  | **Minimum** | **Maximum** |  |
| PC aa C36:4 | 5.52 | 10.84 | 568 |
| PC aa C36:5 | 5.70 | 11.04 | 568 |
| PC aa C36:6 | 3.57 | 16.59 | 568 |
| PC aa C38:0 | 5.80 | 12.78 | 568 |
| PC aa C38:3 | 5.99 | 13.07 | 568 |
| PC aa C38:4 | 5.58 | 10.51 | 568 |
| PC aa C38:5 | 5.84 | 11.95 | 568 |
| PC aa C38:6 | 5.72 | 11.20 | 568 |
| PC aa C40:1 | 5.28 | 6.37 | 294 |
| PC aa C40:2 | 6.49 | 18.11 | 568 |
| PC aa C40:3 | 7.10 | 16.21 | 568 |
| PC aa C40:4 | 6.27 | 11.31 | 568 |
| PC aa C40:5 | 5.86 | 12.05 | 568 |
| PC aa C40:6 | 5.95 | 11.18 | 568 |
| PC aa C42:0 | 7.71 | 14.11 | 568 |
| PC aa C42:1 | 9.00 | 17.61 | 568 |
| PC aa C42:2 | 4.80 | 14.44 | 568 |
| PC aa C42:4 | 7.37 | 15.77 | 568 |
| PC aa C42:5 | 8.57 | 15.84 | 568 |
| PC aa C42:6 | 6.69 | 21.01 | 568 |
| **Acyl-alkylphosphatidylcholines** | |  |  |
| PC ae C30:0 | 6.64 | 12.23 | 568 |
| PC ae C30:2 | 6.19 | 15.98 | 568 |
| PC ae C32:1 | 5.88 | 11.92 | 568 |
| PC ae C32:2 | 5.98 | 12.83 | 568 |
| PC ae C34:0 | 6.63 | 13.26 | 568 |
| PC ae C34:1 | 6.19 | 12.07 | 568 |
| PC ae C34:2 | 5.78 | 12.07 | 568 |
| PC ae C34:3 | 5.93 | 10.80 | 568 |
| PC ae C36:0 | 7.22 | 15.99 | 320 |
| PC ae C36:1 | 6.50 | 12.31 | 320 |
| PC ae C36:2 | 5.34 | 11.39 | 568 |
| PC ae C36:3 | 5.72 | 13.16 | 568 |
| PC ae C36:4 | 6.01 | 12.23 | 568 |
| PC ae C36:5 | 5.76 | 11.66 | 568 |
| PC ae C38:0 | 5.84 | 20.23 | 568 |
| PC ae C38:2 | 6.42 | 15.68 | 568 |
| PC ae C38:3 | 5.29 | 12.27 | 568 |
| PC ae C38:4 | 5.87 | 12.08 | 568 |
| PC ae C38:5 | 6.51 | 11.92 | 568 |
| PC ae C38:6 | 6.06 | 11.46 | 568 |
| PC ae C40:1 | 7.61 | 16.48 | 568 |
| Continues |  |  |  |
| **Table S1 continued** |  |  |  |
| **Metabolites** | **CV^1^** | | **n_QC_^2^** |
|  | **Minimum** | **Maximum** |  |
| PC ae C40:2 | 5.34 | 12.46 | 568 |
| PC ae C40:3 | 5.15 | 13.56 | 568 |
| PC ae C40:4 | 6.06 | 13.91 | 568 |
| PC ae C40:5 | 5.84 | 11.16 | 568 |
| PC ae C40:6 | 5.93 | 11.28 | 568 |
| PC ae C42:1 | 8.10 | 16.02 | 568 |
| PC ae C42:2 | 6.34 | 14.93 | 568 |
| PC ae C42:3 | 5.80 | 14.20 | 568 |
| PC ae C42:4 | 6.53 | 14.32 | 568 |
| PC ae C42:5 | 5.55 | 11.44 | 568 |
| PC ae C44:3 | 12.05 | 22.19 | 568 |
| PC ae C44:4 | 7.81 | 16.86 | 568 |
| PC ae C44:5 | 6.40 | 11.09 | 568 |
| PC ae C44:6 | 6.92 | 13.41 | 568 |
| **HEXOSE** |  |  |  |
| Hexose | 4.86 | 6.53 | 568 |
| **SPHINGOLIPIDS** |  |  |  |
| **Hydroxysphingomyelins** |  |  |  |
| SM (OH) C14:1 | 5.52 | 8.56 | 568 |
| SM (OH) C16:1 | 5.82 | 9.66 | 320 |
| SM (OH) C22:1 | 6.66 | 9.74 | 320 |
| SM (OH) C22:2 | 6.77 | 9.32 | 568 |
| SM (OH) C24:1 | 6.24 | 18.87 | 568 |
| **Sphingomyelins** |  |  |  |
| SM C16:0 | 6.01 | 9.11 | 568 |
| SM C16:1 | 5.55 | 8.64 | 568 |
| SM C18:0 | 6.40 | 8.88 | 568 |
| SM C18:1 | 6.08 | 8.21 | 568 |
| SM C20:2 | 11.13 | 19.84 | 568 |
| SM C24:0 | 5.09 | 9.14 | 568 |
| SM C24:1 | 6.15 | 10.33 | 568 |
| SM C26:0 | 16.62 | 26.34 | 568 |
| SM C26:1 | 10.44 | 18.99 | 568 |

Abbreviations: CV, coefficient of variation; QC, quality control.

^1^ The CVs were calculated across all QC samples in each of five assay rounds. Min and Max provides the range of CVs across the five assay rounds.

^2^ Each analytical batch included four to ten QC samples, amounting to between 8 and 248 QC samples per assay round. The number is the total number of QC samples used to calculate the corresponding CV across all assay rounds.

^3^ Lysine was not excluded from the analysis, although the CV was >20% for one assay rounds. The CV of 22.63% was based on the smallest assay round with only 8 QC samples and thus lysine was thus kept in the analysis.

## Table S2. Loadings for the original metabolites on three treelet components derived using treelet transform at cut-level 97 in 3057 control participants from EPIC

| **Metabolites** | **TC1** | **TC2** | **TC3** |
| --- | --- | --- | --- |
| **ACYLCARNITINES** |  |  |  |
| C0 |  |  |  |
| C2 |  |  |  |
| C3 |  |  |  |
| C14:1 |  |  |  |
| C16 |  |  |  |
| C18 |  |  |  |
| C18:1 |  | 0.3809 |  |
| C18:2 |  | 0.5988 |  |
| **AMINO ACIDS** |  |  |  |
| Alanine |  |  |  |
| Arginine |  |  |  |
| Asparagine |  |  |  |
| Citrulline |  |  |  |
| Glutamate |  | 0.5618 |  |
| Glutamine |  |  |  |
| Glycine |  |  |  |
| Histidine |  |  |  |
| Isoleucine |  |  |  |
| Leucine |  |  |  |
| Lysine |  |  |  |
| Methionine |  |  |  |
| Ornithine |  | 0.2995 |  |
| Phenylalanine |  |  |  |
| Proline |  |  |  |
| Serine |  |  |  |
| t4-hydroxyproline |  |  |  |
| Threonine |  |  |  |
| Tryptophan |  |  |  |
| Tyrosine |  |  |  |
| Valine |  |  |  |
| **BIOGENIC AMINES** |  |  |  |
| ADMA |  |  |  |
| Creatinine |  |  |  |
| Kynurenine |  |  |  |
| Sarcosine |  |  |  |
| Taurine |  | 0.3017 |  |
| **GLYCEROPHOSPHOLIPIDS** |  |  |  |
| **Lysophosphatidylcholines** |  |  |  |
| Lyso PC a C16:0 |  |  | 0.2828 |
| Lyso PC a C16:1 |  |  | 0.3523 |
| Lyso PC a C17:0 |  |  | 0.3241 |
| Lyso PC a C18:0 |  |  | 0.3222 |
| Lyso PC a C18:1 |  |  | 0.3719 |
| Lyso PC a C18:2 |  |  | 0.3390 |

Continues

**Table S2 continued**

| **Metabolites** | **TC1** | **TC2** | **TC3** |
| --- | --- | --- | --- |
| Lyso PC a C20:3 |  |  | 0.4162 |
| Lyso PC a C20:4 |  |  | 0.4008 |
| **Diacyl-phosphatidylcholines** | |  |  |
| PC aa C28:1 | 0.1341 |  |  |
| PC aa C30:0 | 0.1758 |  |  |
| PC aa C32:0 | 0.1006 |  |  |
| PC aa C32:1 | 0.2927 |  |  |
| PC aa C32:3 | 0.1163 |  |  |
| PC aa C34:1 | 0.1342 |  |  |
| PC aa C34:2 | 0.0757 |  |  |
| PC aa C34:3 | 0.1425 |  |  |
| PC aa C34:4 | 0.1575 |  |  |
| PC aa C36:0 | 0.1279 |  |  |
| PC aa C36:1 | 0.1299 |  |  |
| PC aa C36:2 | 0.0774 |  |  |
| PC aa C36:3 | 0.0834 |  |  |
| PC aa C36:4 | 0.1026 |  |  |
| PC aa C36:5 | 0.2418 |  |  |
| PC aa C36:6 | 0.1819 |  |  |
| PC aa C38:0 | 0.1411 |  |  |
| PC aa C38:3 | 0.0977 |  |  |
| PC aa C38:4 | 0.1103 |  |  |
| PC aa C38:5 | 0.1181 |  |  |
| PC aa C38:6 | 0.1373 |  |  |
| PC aa C40:2 | 0.1429 |  |  |
| PC aa C40:3 | 0.1351 |  |  |
| PC aa C40:4 | 0.1105 |  |  |
| PC aa C40:5 | 0.1387 |  |  |
| PC aa C40:6 | 0.1467 |  |  |
| PC aa C42:0 | 0.1042 |  |  |
| PC aa C42:1 | 0.1035 |  |  |
| PC aa C42:2 | 0.1092 |  |  |
| PC aa C42:4 | 0.0625 |  |  |
| PC aa C42:5 | 0.1374 |  |  |
| **Acyl-alkyl-phosphatidylcholines** | |  |  |
| PC ae C30:0 | 0.1692 |  |  |
| PC ae C30:2 | 0.1305 |  |  |
| PC ae C32:1 | 0.0925 |  |  |
| PC ae C32:2 | 0.0985 |  |  |
| PC ae C34:0 | 0.1448 |  |  |
| PC ae C34:1 | 0.1177 |  |  |
| PC ae C34:2 | 0.0976 |  |  |
| PC ae C34:3 | 0.1085 |  |  |
| PC ae C36:0 | 0.1032 |  |  |
| PC ae C36:1 | 0.1283 |  |  |
| PC ae C36:2 | 0.1148 |  |  |
| PC ae C36:3 | 0.0918 |  |  |
| PC ae C36:4 | 0.0973 |  |  |
| PC ae C36:5 | 0.1022 |  |  |
| PC ae C38:2 | 0.1119 |  |  |

Continues

**Table S2 continued**

| **Metabolites** | **TC1** | **TC2** | **TC3** |
| --- | --- | --- | --- |
| PC ae C38:3 | 0.1096 |  |  |
| PC ae C38:4 | 0.0856 |  |  |
| PC ae C38:5 | 0.0799 |  |  |
| PC ae C38:6 | 0.1174 |  |  |
| PC ae C40:1 | 0.1003 |  |  |
| PC ae C40:2 | 0.1222 |  |  |
| PC ae C40:3 | 0.0921 |  |  |
| PC ae C40:4 | 0.0772 |  |  |
| PC ae C40:5 | 0.0725 |  |  |
| PC ae C40:6 | 0.1045 |  |  |
| PC ae C42:1 | 0.0829 |  |  |
| PC ae C42:2 | 0.0997 |  |  |
| PC ae C42:3 | 0.0941 |  |  |
| PC ae C42:4 | 0.1127 |  |  |
| PC ae C42:5 | 0.0716 |  |  |
| PC ae C44:4 | 0.0918 |  |  |
| PC ae C44:5 | 0.0924 |  |  |
| PC ae C44:6 | 0.0952 |  |  |
| **HEXOSE** |  |  |  |
| Hexose |  |  |  |
| **SPHINGOLIPIDS** |  |  |  |
| **Hydroxysphingomyelins** |  |  |  |
| SM (OH) C14:1 | 0.1206 |  |  |
| SM (OH) C16:1 | 0.1116 |  |  |
| SM (OH) C22:1 |  |  |  |
| SM (OH) C22:2 | 0.0979 |  |  |
| SM (OH) C24:1 |  |  |  |
| **Sphingomyelins** |  |  |  |
| SM C16:0 |  |  |  |
| SM C16:1 |  |  |  |
| SM C18:0 |  |  |  |
| SM C18:1 |  |  |  |
| SM C20:2 |  |  |  |
| SM C24:0 |  |  |  |
| SM C24:1 |  |  |  |
| Variance explained, % | 21.5 | 5.2 | 4.7 |

Abbreviations: EPIC, European Prospective Investigation into Cancer and Nutrition; TC, treelet component

## Table S3. Correlations between treelet component scores in 3057 control participants in EPIC

|  | **TC1** | **TC2** | **TC3** |
| --- | --- | --- | --- |
| **TC1, phosphatidylcholines and hydroxyl sphingomyelins** | 1 |  |  |
| **TC2, acylcarnitines, glutamate, ornithine and taurine** | -0.07 | 1 |  |
| **TC3, lysophosphatidylcholines** | 0.46 | 0.17 | 1 |

Abbreviations: EPIC, European Prospective Investigation into Cancer and Nutrition; TC, treelet component

Table S4. Risk of prostate cancer in relation to treelet component scores in 3057 matched case-control sets from EPIC^1^

|  | **n_case_** | **OR (95% CI) for quintiles of treelet component score** | | | | | **1 standard deviation increase** | | |
| --- | --- | --- | --- | --- | --- | --- | --- | --- | --- |
|  |  | **1** | **2** | **3** | **4** | **5** | **OR (95% CI)** | **p** | **p-het** |
| **TC1** |  |  |  |  |  |  |  |  |  |
| Overall prostate cancer | 3057 | Ref | 1.03 (0.87 to 1.21) | 0.98 (0.83 to 1.16) | 1.04 (0.87 to 1.24) | 0.82 (0.68 to 1.00) | 0.94 (0.89 to 1.00) | 0.069 |  |
| Stage |  |  |  |  |  |  |  |  |  |
| Localised^2^ | 1306 | Ref | 1.16 (0.89 to 1.50) | 1.21 (0.94 to 1.58) | 1.31 (0.99 to 1.73) | 1.13 (0.84 to 1.52) | 1.03 (0.93 to 1.13) | 0.567 |  |
| Advanced^3^ | 580 | Ref | 0.77 (0.51 to 1.18) | 0.70 (0.46 to 1.06) | 0.63 (0.40 to 0.98) | 0.47 (0.29 to 0.75) | 0.77 (0.66 to 0.89) | 0.0007 | 0.002 |
| Follow-up ≤10 yrs | 328 | Ref | 0.67 (0.35 to 1.30) | 0.64 (0.34 to 1.20) | 0.65 (0.34 to 1.26) | 0.39 (0.20 to 0.77) | 0.76 (0.62 to 0.93) | 0.009 |  |
| Follow-up >10 yrs | 252 | Ref | 0.78 (0.50 to 1.24) | 0.74 (0.47 to 1.18) | 0.67 (0.41 to 1.10) | 0.51 (0.29 to 0.87) | 0.79 (0.62 to 1.00) | 0.049 | 0.865 |
| Non-aggressive^4^ | 1519 | Ref | 1.09 (0.86 to 1.38) | 1.09 (0.86 to 1.39) | 1.14 (0.88 to 1.48) | 1.03 (0.78 to 1.36) | 0.99 (0.90 to 1.08) | 0.758 |  |
| Aggressive^5^ | 367 | Ref | 0.76 (0.44 to 1.30) | 0.79 (0.46 to 1.36) | 0.70 (0.40 to 1.24) | 0.44 (0.24 to 0.79) | 0.79 (0.66 to 0.95) | 0.013 | 0.018 |
| Follow-up ≤10 yrs | 229 | Ref | 0.86 (0.37 to 1.96) | 1.06 (0.51 to 2.23) | 1.03 (0.48 to 2.25) | 0.46 (0.21 to 1.00) | 0.76 (0.60 to 0.97) | 0.026 |  |
| Follow-up >10 yrs^6^ | 137 | Ref | 0.65 (0.30 to 1.41) | 0.46 ( 0.19 to 1.12) | 0.35 (0.14 to 0.89) | 0.44 (0.15 to 1.32) | 0.83 (0.61 to 1.13) | 0.235 | 0.749 |
| Grade |  |  |  |  |  |  |  |  |  |
| Low-intermediate^7^ | 2157 | Ref | 1.01 (0.83 to 1.23) | 1.01 (0.83 to 1.23) | 1.10 (0.89 to 1.37) | 0.87 (0.69 to 1.10) | 0.96 (0.89 to 1.03) | 0.259 |  |
| High^8^ | 317 | Ref | 1.05 (0.61 to 1.81) | 1.05 (0.60 to 1.81) | 1.03 (0.58 to 1.81) | 0.66 (0.35 to 1.24) | 0.87 (0.71 to 1.07) | 0.188 | 0.562 |
| Prostate cancer death^9^ | 297 | Ref | 0.89 (0.48 to 1.63) | 0.86 (0.48 to 1.55) | 0.88 (0.45 to 1.70) | 0.61 (0.31 to 1.18) | 0.99 (0.80 to 1.21) | 0.903 |  |
|  |  |  |  |  |  |  |  |  |  |
| **TC2** |  |  |  |  |  |  |  |  |  |
| Overall prostate cancer | 3057 | Ref | 0.95 (0.81 to 1.12) | 0.91 (0.76 to 1.09) | 0.92 (0.76 to 1.11) | 0.97 (0.77 to 1.21) | 0.98 (0.90 to 1.07) | 0.679 |  |
| Stage |  |  |  |  |  |  |  |  |  |
| Localised^2^ | 1306 | Ref | 0.86 (0.68 to 1.10) | 0.82 (0.63 to 1.06) | 0.86 (0.65 to 1.14) | 0.96 (0.69 to 1.35) | 1.01 (0.88 to 1.16) | 0.847 |  |
| Advanced^3^ | 580 | Ref | 0.98 (0.66 to 1.48) | 0.71 (0.46 to 1.10) | 0.67 (0.42 to 1.06) | 0.55 (0.32 to 0.95) | 0.72 (0.57 to 0.90) | 0.005 | 0.058 |
| Follow-up ≤10 yrs | 328 | Ref | 0.73 (0.42 to 1.27) | 0.67 (0.37 to 1.23) | 0.67 (0.36 to 1.25) | 0.40 (0.18 to 0.88) | 0.73 (0.54 to 0.99) | 0.041 |  |
| Follow-up >10 yrs | 252 | Ref | 1.11 (0.70 to 1.76) | 0.73 (0.44 to 1.20) | 0.71 (0.41 to 1.24) | 0.58 (0.32 to 1.06) | 0.70 (0.48 to 1.02) | 0.066 | 0.475 |
| Non-aggressive^4^ | 1519 | Ref | 0.93 (0.75 to 1.16) | 0.85 (0.67 to 1.08) | 0.89 (0.68 to 1.16) | 0.94 (0.69 to 1.30) | 0.99 (0.87 to 1.13) | 0.869 |  |
| Aggressive^5^ | 367 | Ref | 0.84 (0.50 to 1.43) | 0.61 (0.35 to 1.07) | 0.59 (0.34 to 1.04) | 0.52 (0.27 to 0.99) | 0.70 (0.53 to 0.93) | 0.014 | 0.079 |
| Follow-up ≤10 yrs | 229 | Ref | 0.55 (0.28 to 1.10) | 0.45 (0.21 to 0.97) | 0.50 (0.23 to 1.06) | 0.33 (0.13 to 0.80) | 0.66 (0.46 to 0.96) | 0.030 |  |
| Follow-up >10 yrs^6^ | 137 | Ref | 1.48 (0.59 to 3.70) | 0.88 (0.36 to 2.15) | 0.64 (0.25 to 1.61) | 0.97 (0.35 to 2.67) | 0.74 (0.47 to 1.17) | 0.194 | 0.695 |
| Grade |  |  |  |  |  |  |  |  |  |
| Low-intermediate^7^ | 2157 | Ref | 0.94 (0.77 to 1.13) | 0.89 (0.72 to 1.10) | 0.90 (0.72 to 1.12) | 0.88 (0.68 to 1.16) | 0.93 (0.84 to 1.04) | 0.219 |  |
| High^8^ | 317 | Ref | 0.89 (0.48 to 1.67) | 0.61 (0.34 to 1.11) | 0.83 (0.44 to 1.59) | 1.03 (0.50 to 2.12) | 1.03 (0.77 to 1.38) | 0.831 | 0.369 |
| Prostate cancer death^9^ | 297 | Ref | 1.00 (0.51 to 1.96) | 0.93 (0.48 to 1.81) | 1.05 (0.54 to 2.04) | 0.96 (0.45 to 2.02) | 1.03 (0.78 to 1.37) | 0.828 |  |

Continues

**Table S4** continued

|  | **n_case_** | **OR (95% CI) for quintiles of treelet component score** | | | | | **1 standard deviation increase** | | |
| --- | --- | --- | --- | --- | --- | --- | --- | --- | --- |
|  |  | **1** | **2** | **3** | **4** | **5** | **OR (95% CI)** | **p** | **p-het** |
| **TC3** |  |  |  |  |  |  |  |  |  |
| Overall prostate cancer | 3057 | Ref | 0.93 (0.80 to 1.10) | 0.97 (0.82 to 1.15) | 0.82 (0.68 to 0.98) | 0.84 (0.69 to 1.02) | 0.94 (0.88 to 1.01) | 0.073 |  |
| Stage |  |  |  |  |  |  |  |  |  |
| Localised^2^ | 1306 | Ref | 0.99 (0.78 to 1.27) | 1.13 (0.87 to 1.46) | 1.02 (0.77 to 1.35) | 0.94 (0.70 to 1.28) | 0.99 (0.90 to 1.10) | 0.919 |  |
| Advanced^3^ | 580 | Ref | 0.59 (0.40 to 0.87) | 0.59 (0.39 to 0.90) | 0.49 (0.31 to 0.76) | 0.56 (0.35 to 0.91) | 0.81 (0.69 to 0.95) | 0.009 | 0.012 |
| Follow-up ≤10 yrs | 328 | Ref | 0.69 (0.38 to 1.25) | 0.65 (0.35 to 1.19) | 0.55 (0.30 to 1.02) | 0.65 (0.34 to 1.26) | 0.86 (0.69 to 1.08) | 0.199 |  |
| Follow-up >10 yrs | 252 | Ref | 0.61 (0.40 to 0.93) | 0.66 (0.42 to 1.03) | 0.45 (0.28 to 0.74) | 0.60 (0.35 to 1.02) | 0.76 (0.60 to 0.97) | 0.030 | 0.643 |
| Non-aggressive^4^ | 1519 | Ref | 0.97 (0.77 to 1.22) | 0.98 (0.77 to 1.24) | 0.93 (0.71 to 1.20) | 0.89 (0.67 to 1.18) | 0.97 (0.88 to 1.06) | 0.465 |  |
| Aggressive^5^ | 367 | Ref | 0.47 (0.28 to 0.79) | 0.76 (0.46 to 1.25) | 0.45 (0.26 to 0.79) | 0.54 (0.30 to 0.98) | 0.83 (0.69 to 1.01) | 0.067 | 0.045 |
| Follow-up ≤10 yrs | 229 | Ref | 0.46 (0.21 to 0.97) | 0.62 (0.30 to 1.26) | 0.42 (0.20 to 0.90) | 0.49 (0.22 to 1.08) | 0.80 (0.61 to 1.04) | 0.097 |  |
| Follow-up >10 yrs^6^ | 137 | Ref | 0.47 (0.21 to 1.05) | 1.15 (0.53 to 2.48) | 0.45 (0.17 to 1.18) | 0.73 (0.27 to 1.99) | 0.93 (0.68 to 1.27) | 0.661 | 0.501 |
| Grade |  |  |  |  |  |  |  |  |  |
| Low-intermediate^7^ | 2157 | Ref | 0.95 (0.79 to 1.16) | 1.07 (0.88 to 1.31) | 0.91 (0.73 to 1.13) | 0.89 (0.70 to 1.13) | 0.95 (0.88 to 1.03) | 0.235 |  |
| High^8^ | 317 | Ref | 0.92 (0.54 to 1.56) | 1.10 (0.63 to 1.93) | 0.83 (0.47 to 1.47) | 0.70 (0.36 to 1.33) | 0.92 (0.75 to 1.14) | 0.450 | 0.881 |
| Prostate cancer death^9^ | 297 | Ref | 0.80 (0.45 to 1.43) | 0.56 (0.29 to 1.07) | 0.54 (0.29 to 1.01) | 0.50 (0.25 to 1.00) | 0.77 (0.61 to 0.96) | 0.023 |  |

Abbreviations: EPIC, European Prospective Investigation into Cancer and Nutrition; OR, odds ratio; CI confidence interval; p-het, p value for heterogeneity; TC, treelet component; yrs, years.

^1^ All analyses were matched on centre, length of follow-up, and age (±6 months), time of day (±1 hour) and fasting status (<3, 3–6, >6 hours) at blood collection and further adjusted for exact age at blood collection (continuously), and baseline values for body mass index (quartiles; unknown), smoking (never; past; current; unknown), alcohol intake (<10; 10-19; 20-39; ≥40 g of alcohol per day; unknown), education (primary; secondary; degree level; unknown) and marital status (married or cohabiting; not married or cohabiting; unknown).

^2^ Tumour-node-metastasis score ≤T2 and N0/x and M0, or coded as localised.

^3^ Tumour-node-metastasis score T3-4 and/or N1-3 and/or M1, or coded as advanced.

^4^ Tumour-node-metastasis score ≤T3 and N0/x and M0.

^5^ Tumour-node-metastasis score T4 and/or N1-3 and/or M1.

^6^ One matched set was excluded from this analysis; the case was the only individual with unknown smoking status in this sub-group and the model was thus not stable.

^7^ Gleason score <8 or coded as well, moderately or poorly differentiated.

^8^ Gleason score ≥8 or coded as undifferentiated.

^9^ Matched sets in which the control died, emigrated or was lost to follow-up before the case died were excluded (n=29).

Table S5. Risk of prostate cancer in relation to treelet component scores for TC1 and TC3 mutually adjusted, in 3057 matched case-control sets from EPIC^1^

|  | **n_case_** | **OR (95% CI) for quintiles of treelet component score** | | | | | **1 standard deviation increase** | | |
| --- | --- | --- | --- | --- | --- | --- | --- | --- | --- |
|  |  | **1** | **2** | **3** | **4** | **5** | **OR (95% CI)** | **p** | **p-het** |
| **TC1** |  |  |  |  |  |  |  |  |  |
| Overall prostate cancer | 3057 | Ref | 1.04 (0.88 to 1.23) | 1.00 (0.85 to 1.19) | 1.07 (0.89 to 1.29) | 0.86 (0.70 to 1.06) | 0.96 (0.90 to 1.03) | 0.251 |  |
| Stage |  |  |  |  |  |  |  |  |  |
| Localised^2^ | 1306 | Ref | 1.16 (0.90 to 1.51) | 1.23 (0.94 to 1.61) | 1.33 (0.99 to 1.78) | 1.16 (0.84 to 1.61) | 1.04 (0.93 to 1.15) | 0.493 |  |
| Advanced^3^ | 580 | Ref | 0.82 (0.54 to 1.25) | 0.75 (0.48 to 1.16) | 0.70 (0.44 to 1.11) | 0.54 (0.32 to 0.90) | 0.81 (0.68 to 0.95) | 0.012 | 0.002 |
| Follow-up ≤10 yrs | 328 | Ref | 0.67 (0.35 to 1.31) | 0.64 (0.34 to 1.21) | 0.66 (0.34 to 1.29) | 0.40 (0.20 to 0.82) | 0.76 (0.60 to 0.96) | 0.021 |  |
| Follow-up >10 yrs | 252 | Ref | 1.05 (0.59 to 1.88) | 0.87 (0.46 to 1.66) | 0.68 (0.34 to 1.35) | 0.83 (0.36 to 1.94) | 0.86 (0.66 to 1.11) | 0.248 | 0.829 |
| Non-aggressive^4^ | 1519 | Ref | 1.11 (0.87 to 1.41) | 1.12 (0.87 to 1.44) | 1.18 (0.90 to 1.55) | 1.09 (0.81 to 1.47) | 1.00 (0.91 to 1.11) | 0.987 |  |
| Aggressive^5^ | 367 | Ref | 0.78 (0.45 to 1.35) | 0.82 (0.47 to 1.43) | 0.75 (0.42 to 1.34) | 0.48 (0.26 to 0.90) | 0.82 (0.67 to 1.01) | 0.061 | 0.018 |
| Follow-up ≤10 yrs | 229 | Ref | 0.86 (0.38 to 1.98) | 1.08 (0.51 to 2.27) | 1.08 (0.49 to 2.37) | 0.50 (0.22 to 1.13) | 0.80 (0.61 to 1.04) | 0.090 |  |
| Follow-up >10 yrs^6^ | 137 | Ref | 0.63 (0.29 to 1.39) | 0.44 (0.17 to 1.11) | 0.33 (0.12 to 0.89) | 0.42 (0.14 to 1.30) | 0.83 (0.59 to 1.16) | 0.268 | 0.738 |
| Grade |  |  |  |  |  |  |  |  |  |
| Low-intermediate^7^ | 2157 | Ref | 1.03 (0.84 to 1.25) | 1.03 (0.84 to 1.26) | 1.13 (0.90 to 1.41) | 0.91 (0.71 to 1.16) | 0.97 (0.89 to 1.05) | 0.487 |  |
| High^8^ | 317 | Ref | 1.07 (0.62 to 1.86) | 1.08 (0.61 to 1.90) | 1.08 (0.58 to 2.01) | 0.70 (0.35 to 1.40) | 0.88 (0.69 to 1.11) | 0.277 | 0.576 |
| Prostate cancer death^9^ | 297 | Ref | 0.91 (0.49 to 1.69) | 0.98 (0.53 to 1.81) | 1.06 (0.53 to 2.12) | 0.83 (0.39 to 1.76) | 1.17 (0.92 to 1.50) | 0.205 |  |
|  |  |  |  |  |  |  |  |  |  |
| **TC3** |  |  |  |  |  |  |  |  |  |
| Overall prostate cancer | 3057 | Ref | 0.95 (0.81 to 1.11) | 1.00 (0.84 to 1.19) | 0.85 (0.70 to 1.02) | 0.88 (0.71 to 1.09) | 0.96 (0.89 to 1.03) | 0.271 |  |
| Stage |  |  |  |  |  |  |  |  |  |
| Localised^2^ | 1306 | Ref | 0.98 (0.76 to 1.26) | 1.10 (0.84 to 1.43) | 0.99 (0.74 to 1.32) | 0.90 (0.64 to 1.25) | 0.98 (0.87 to 1.09) | 0.695 |  |
| Advanced^3^ | 580 | Ref | 0.62 (0.42 to 0.93) | 0.67 (0.44 to 1.03) | 0.59 (0.37 to 0.93) | 0.74 (0.44 to 1.23) | 0.89 (0.75 to 1.07) | 0.224 | 0.012 |
| Follow-up ≤10 yrs | 328 | Ref | 0.75 (0.41 to 1.36) | 0.75 (0.40 to 1.40) | 0.68 (0.36 to 1.29) | 0.90 (0.44 to 1.84) | 0.99 (0.77 to 1.27) | 0.929 |  |
| Follow-up >10 yrs | 252 | Ref | 0.50 (0.28 to 0.89) | 0.64 (0.35 to 1.17) | 0.47 (0.22 to 0.99) | 0.59 (0.25 to 1.35) | 0.82 (0.62 to 1.07) | 0.141 | 0.584 |
| Non-aggressive^4^ | 1519 | Ref | 0.97 (0.77 to 1.22) | 0.98 (0.76 to 1.25) | 0.92 (0.71 to 1.21) | 0.89 (0.65 to 1.21) | 0.97 (0.87 to 1.07) | 0.507 |  |
| Aggressive^5^ | 367 | Ref | 0.48 (0.28 to 0.82) | 0.85 (0.51 to 1.42) | 0.52 (0.30 to 0.93) | 0.70 (0.37 to 1.32) | 0.91 (0.73 to 1.13) | 0.397 | 0.045 |
| Follow-up ≤10 yrs | 229 | Ref | 0.48 (0.22 to 1.02) | 0.70 (0.34 to 1.45) | 0.49 (0.23 to 1.05) | 0.66 (0.28 to 1.53) | 0.89 (0.66 to 1.20) | 0.448 |  |
| Follow-up >10 yrs^6^ | 137 | Ref | 0.48 (0.21 to 1.09) | 1.29 (0.58 to 2.87) | 0.54 (0.19 to 1.51) | 0.88 (0.30 to 2.56) | 1.01 (0.72 to 1.41) | 0.969 | 0.571 |
| Grade |  |  |  |  |  |  |  |  |  |
| Low-intermediate^7^ | 2157 | Ref | 0.96 (0.79 to 1.17) | 1.09 (0.89 to 1.34) | 0.93 (0.74 to 1.17) | 0.92 (0.72 to 1.19) | 0.97 (0.89 to 1.05) | 0.431 |  |
| High^8^ | 317 | Ref | 0.97 (0.56 to 1.67) | 1.21 (0.67 to 2.20) | 0.95 (0.50 to 1.80) | 0.83 (0.39 to 1.73) | 0.99 (0.77 to 1.26) | 0.925 | 0.909 |
| Prostate cancer death^9^ | 297 | Ref | 0.76 (0.42 to 1.38) | 0.52 (0.27 to 1.02) | 0.49 (0.25 to 0.95) | 0.43 (0.20 to 0.93) | 0.70 (0.54 to 0.92) | 0.010 |  |

Abbreviations: EPIC, European Prospective Investigation into Cancer and Nutrition; OR, odds ratio; CI confidence interval; p-het, p value for heterogeneity; TC, treelet component; yrs, years.

^1^ All analyses were matched on centre, length of follow-up, and age (±6 months), time of day (±1 hour) and fasting status (<3, 3–6, >6 hours) at blood collection and further adjusted for exact age at blood collection (continuously), and baseline values for body mass index (quartiles; unknown), smoking (never; past; current; unknown), alcohol intake (<10; 10-19; 20-39; ≥40 g of alcohol per day; unknown), education (primary; secondary; degree level; unknown) and marital status (married or cohabiting; not married or cohabiting; unknown). TC1 was furthermore adjusted for TC3 (continuously), while TC3 was additionally adjusted for TC1 (continuously).

^2^ Tumour-node-metastasis score ≤T2 and N0/x and M0, or coded as localised.

^3^ Tumour-node-metastasis score T3-4 and/or N1-3 and/or M1, or coded as advanced.

^4^ Tumour-node-metastasis score ≤T3 and N0/x and M0.

^5^ Tumour-node-metastasis score T4 and/or N1-3 and/or M1.

^6^ One matched set was excluded from this analysis; the case was the only individual with unknown smoking status in this sub-group and the model was thus not stable.

^7^ Gleason score <8 or coded as well, moderately or poorly differentiated.

^8^ Gleason score ≥8 or coded as undifferentiated.

^9^ Matched sets in which the control died, emigrated or was lost to follow-up before the case died were excluded (n=29).

**Table S6. Risk of overall prostate cancer in relation to treelet component scores, in 2018 matched case-control sets from EPIC^1^**

| **Treelet components** | **n_case_** | **OR (95% CI) for quintiles of treelet component score** | | | | | **1 standard deviation increase** | |
| --- | --- | --- | --- | --- | --- | --- | --- | --- |
|  |  | **1** | **2** | **3** | **4** | **5** | **OR (95% CI)** | **p** |
| TC1 | 2018 | Ref | 0.95 (0.79 to 1.15) | 0.89 (0.73 to 1.08) | 0.93 (0.75 to 1.16) | 0.71 (0.55 to 0.91) | 0.90 (0.83 to 0.98) | 0.011 |
| TC2 | 2018 | Ref | 1.03 (0.84 to 1.27) | 1.05 (0.84 to 1.31) | 1.01 (0.79 to 1.28) | 1.05 (0.80 to 1.38) | 1.01 (0.90 to 1.13) | 0.909 |
| TC3 | 2018 | Ref | 0.93 (0.80 to 1.15) | 1.03 (0.84 to 1.25) | 0.80 (0.64 to 1.00) | 0.84 (0.65 to 1.07) | 0.94 (0.87 to 1.02) | 0.149 |

Abbreviations: EPIC, European Prospective Investigation into Cancer and Nutrition; OR, odds ratio; CI confidence interval; TC, treelet component.

^1^ The analysis was restricted to 2018 cases and their matched controls, who were not included in our previous publication [1]. All analyses were matched on centre, length of follow-up, and age (±6 months), time of day (±1 hour) and fasting status (<3, 3–6, >6 hours) at blood collection and further adjusted for exact age at blood collection (continuously), and baseline values for body mass index (quartiles; unknown), smoking (never; past; current; unknown), alcohol intake (<10; 10-19; 20-39; ≥40 g of alcohol per day; unknown), education (primary; secondary; degree level; unknown) and marital status (married or cohabiting; not married or cohabiting; unknown).

## Table S7. Loadings for the original metabolites on nine principal components derived using principal component analysis in 3057 control participants from EPIC

| **Metabolites** | **PC1** | **PC2** | **PC3** | **PC4** | **PC5** | **PC6** | **PC7** | **PC8** | **PC9** |
| --- | --- | --- | --- | --- | --- | --- | --- | --- | --- |
| **ACYLCARNITINES** |  |  |  |  |  |  |  |  |  |
| C0 | 0.027 | -0.001 | -0.036 | 0.067 | 0.035 | 0.014 | 0.045 | 0.066 | 0.015 |
| C2 | 0.037 | -0.020 | -0.042 | 0.096 | 0.038 | -0.199 | 0.127 | 0.155 | 0.094 |
| C3 | 0.000 | 0.005 | -0.060 | 0.122 | 0.130 | 0.167 | 0.307 | 0.092 | 0.056 |
| C14:1 | 0.005 | -0.034 | 0.001 | 0.225 | 0.067 | -0.425 | 0.398 | 0.197 | 0.179 |
| C16 | 0.054 | 0.098 | -0.156 | 0.018 | 0.009 | -0.129 | 0.133 | -0.001 | 0.035 |
| C18 | 0.057 | 0.140 | -0.108 | -0.019 | 0.085 | -0.105 | 0.210 | 0.042 | 0.104 |
| C18:1 | 0.012 | 0.247 | -0.110 | 0.140 | 0.024 | -0.197 | 0.123 | -0.044 | 0.036 |
| C18:2 | -0.030 | 0.399 | -0.067 | 0.113 | 0.056 | -0.088 | 0.052 | 0.163 | -0.074 |
| **AMINO ACIDS** |  |  |  |  |  |  |  |  |  |
| Alanine | 0.002 | 0.063 | -0.066 | 0.012 | 0.005 | 0.139 | -0.008 | 0.004 | -0.035 |
| Arginine | -0.003 | -0.358 | 0.202 | 0.054 | 0.083 | 0.183 | 0.053 | 0.207 | 0.120 |
| Asparagine | -0.004 | 0.078 | 0.014 | 0.019 | 0.049 | 0.118 | 0.005 | -0.008 | 0.035 |
| Citrulline | 0.005 | 0.059 | 0.010 | -0.006 | 0.020 | 0.041 | 0.001 | 0.084 | 0.045 |
| Glutamate | -0.007 | 0.314 | -0.238 | 0.061 | -0.089 | 0.119 | 0.075 | -0.040 | -0.209 |
| Glutamine | 0.030 | -0.005 | 0.040 | -0.010 | 0.051 | 0.046 | -0.032 | 0.045 | 0.035 |
| Glycine | 0.002 | 0.099 | 0.023 | -0.019 | 0.052 | 0.073 | -0.008 | -0.010 | 0.047 |
| Histidine | 0.004 | 0.063 | -0.020 | 0.039 | 0.041 | 0.089 | 0.028 | 0.000 | 0.021 |
| Isoleucine | -0.005 | 0.026 | -0.049 | 0.100 | 0.065 | 0.222 | 0.076 | 0.043 | 0.022 |
| Leucine | -0.002 | 0.040 | -0.045 | 0.082 | 0.051 | 0.188 | 0.080 | 0.033 | -0.005 |
| Lysine | -0.002 | 0.041 | -0.011 | 0.085 | 0.039 | 0.147 | 0.088 | 0.002 | 0.004 |
| Methionine | -0.004 | -0.014 | 0.008 | 0.088 | 0.075 | 0.202 | 0.048 | 0.046 | 0.036 |
| Ornithine | -0.002 | 0.235 | -0.084 | 0.049 | 0.060 | 0.146 | 0.059 | -0.029 | -0.021 |
| Phenylalanine | 0.000 | 0.055 | -0.030 | 0.037 | 0.037 | 0.126 | 0.057 | 0.029 | -0.016 |
| Proline | 0.011 | 0.053 | -0.072 | -0.012 | 0.052 | 0.210 | 0.003 | 0.028 | -0.018 |
| Serine | -0.009 | 0.100 | 0.044 | 0.026 | 0.065 | 0.079 | 0.028 | -0.021 | 0.055 |
| t4-hydroxyproline | 0.010 | -0.044 | 0.014 | 0.154 | 0.143 | 0.209 | 0.094 | 0.156 | 0.180 |
| Threonine | -0.011 | 0.055 | 0.018 | 0.055 | 0.060 | 0.135 | 0.008 | -0.012 | 0.041 |
| Tryptophan | 0.010 | -0.003 | -0.026 | 0.047 | 0.048 | 0.110 | 0.042 | -0.011 | -0.010 |
| Tyrosine | 0.005 | 0.022 | -0.062 | 0.071 | 0.042 | 0.178 | 0.062 | 0.033 | -0.047 |
| Valine | -0.006 | 0.012 | -0.036 | 0.078 | 0.031 | 0.155 | 0.084 | 0.009 | -0.007 |
| **BIOGENIC AMINES** |  |  |  |  |  |  |  |  |  |
| ADMA | -0.009 | 0.071 | -0.030 | 0.033 | 0.017 | 0.049 | 0.034 | 0.019 | -0.009 |
| Creatinine | 0.015 | 0.023 | 0.004 | 0.001 | 0.011 | 0.039 | 0.038 | 0.024 | 0.020 |
| Kynurenine | 0.011 | 0.017 | -0.036 | -0.010 | 0.029 | 0.055 | 0.099 | 0.034 | -0.062 |
| Sarcosine | 0.003 | -0.004 | -0.051 | 0.161 | 0.101 | 0.287 | -0.038 | 0.045 | 0.059 |
| Taurine | 0.011 | 0.226 | -0.041 | 0.027 | -0.039 | 0.066 | 0.031 | -0.026 | -0.085 |
| **GLYCEROPHOSPHOLIPIDS** | |  |  |  |  |  |  |  |  |
| **Lysophosphatidylcholines** | |  |  |  |  |  |  |  |  |
| Lyso PC a C16:0 | 0.070 | 0.107 | -0.067 | 0.065 | 0.087 | -0.020 | -0.124 | -0.098 | 0.095 |
| Lyso PC a C16:1 | 0.114 | 0.073 | -0.194 | -0.004 | 0.032 | -0.033 | -0.135 | -0.025 | 0.135 |
| Lyso PC a C17:0 | 0.093 | 0.151 | 0.054 | -0.082 | 0.103 | 0.024 | -0.039 | -0.239 | 0.175 |
| Lyso PC a C18:0 | 0.071 | 0.124 | 0.008 | 0.068 | 0.118 | -0.030 | -0.144 | -0.076 | 0.123 |
| Lyso PC a C18:1 | 0.086 | 0.070 | -0.019 | 0.076 | 0.146 | -0.089 | -0.168 | -0.163 | 0.239 |
| Lyso PC a C18:2 | 0.065 | 0.078 | 0.067 | -0.042 | 0.188 | 0.045 | -0.197 | -0.007 | 0.298 |
| Lyso PC a C20:3 | 0.098 | -0.021 | -0.128 | 0.038 | 0.214 | -0.077 | -0.168 | -0.084 | 0.185 |
| Lyso PC a C20:4 | 0.075 | 0.010 | -0.017 | 0.122 | 0.204 | -0.108 | -0.202 | -0.078 | 0.169 |
| **Diacyl-phosphatidylcholines** | |  |  |  |  |  |  |  |  |
| PC aa C28:1 | 0.133 | -0.023 | -0.023 | -0.133 | 0.003 | 0.032 | 0.094 | -0.056 | -0.012 |
| PC aa C30:0 | 0.161 | -0.098 | -0.164 | -0.081 | -0.025 | 0.064 | 0.014 | -0.058 | -0.061 |
| PC aa C32:0 | 0.114 | -0.044 | -0.061 | 0.026 | 0.018 | -0.010 | 0.003 | -0.002 | -0.060 |
| PC aa C32:1 | 0.201 | -0.206 | -0.393 | -0.024 | -0.060 | -0.011 | -0.022 | 0.057 | -0.066 |
| Continues |  |  |  |  |  |  |  |  |  |
| **Table S7 continued** |  |  |  |  |  |  |  |  |  |
| **Metabolites** | **PC1** | **PC2** | **PC3** | **PC4** | **PC5** | **PC6** | **PC7** | **PC8** | **PC9** |
| PC aa C32:3 | 0.128 | 0.043 | 0.004 | -0.136 | -0.049 | 0.052 | 0.033 | 0.094 | 0.071 |
| PC aa C34:1 | 0.121 | -0.094 | -0.135 | 0.067 | 0.023 | -0.061 | 0.000 | -0.056 | -0.013 |
| PC aa C34:2 | 0.082 | -0.006 | -0.023 | -0.024 | 0.042 | 0.023 | -0.039 | 0.091 | 0.005 |
| PC aa C34:3 | 0.143 | -0.015 | -0.155 | -0.144 | -0.032 | 0.047 | -0.032 | 0.157 | 0.048 |
| PC aa C34:4 | 0.149 | -0.098 | -0.168 | 0.000 | 0.011 | 0.059 | -0.051 | 0.068 | -0.017 |
| PC aa C36:0 | 0.097 | 0.005 | 0.146 | 0.151 | -0.093 | 0.043 | 0.012 | -0.029 | 0.071 |
| PC aa C36:1 | 0.118 | -0.071 | -0.095 | 0.071 | 0.038 | -0.029 | 0.028 | -0.067 | -0.010 |
| PC aa C36:2 | 0.081 | 0.002 | 0.001 | 0.002 | 0.064 | 0.026 | -0.036 | 0.085 | 0.014 |
| PC aa C36:3 | 0.094 | -0.011 | -0.085 | -0.015 | 0.079 | -0.003 | -0.027 | 0.072 | -0.019 |
| PC aa C36:4 | 0.090 | -0.072 | -0.077 | 0.089 | 0.099 | -0.040 | -0.051 | 0.062 | -0.065 |
| PC aa C36:5 | 0.202 | -0.066 | -0.069 | 0.157 | -0.370 | 0.058 | 0.032 | -0.128 | 0.146 |
| PC aa C36:6 | 0.177 | -0.033 | -0.028 | 0.106 | -0.262 | 0.097 | 0.028 | -0.083 | 0.110 |
| PC aa C38:0 | 0.095 | 0.037 | 0.201 | 0.190 | -0.088 | -0.003 | -0.004 | -0.039 | 0.027 |
| PC aa C38:3 | 0.090 | -0.052 | -0.113 | 0.064 | 0.060 | -0.019 | 0.011 | 0.042 | -0.134 |
| PC aa C38:4 | 0.078 | -0.057 | -0.052 | 0.120 | 0.108 | -0.058 | -0.034 | 0.085 | -0.107 |
| PC aa C38:5 | 0.136 | -0.023 | -0.074 | 0.064 | -0.081 | -0.026 | -0.022 | 0.002 | 0.017 |
| PC aa C38:6 | 0.114 | -0.006 | 0.053 | 0.198 | -0.184 | -0.005 | 0.030 | -0.063 | 0.035 |
| PC aa C40:2 | 0.124 | 0.105 | 0.097 | -0.023 | -0.145 | 0.026 | -0.094 | 0.199 | 0.046 |
| PC aa C40:3 | 0.129 | 0.081 | 0.037 | -0.040 | -0.130 | 0.006 | -0.067 | 0.183 | 0.033 |
| PC aa C40:4 | 0.098 | -0.044 | -0.130 | 0.036 | 0.103 | -0.051 | -0.060 | 0.135 | -0.134 |
| PC aa C40:5 | 0.133 | -0.033 | -0.159 | -0.002 | -0.036 | -0.022 | -0.032 | 0.075 | -0.096 |
| PC aa C40:6 | 0.110 | 0.006 | 0.038 | 0.203 | -0.198 | 0.006 | 0.035 | -0.029 | -0.037 |
| PC aa C42:0 | 0.088 | 0.094 | 0.187 | 0.058 | -0.066 | 0.001 | -0.050 | 0.033 | -0.135 |
| PC aa C42:1 | 0.081 | 0.086 | 0.185 | 0.099 | -0.044 | 0.012 | -0.076 | 0.059 | -0.127 |
| PC aa C42:2 | 0.079 | 0.071 | 0.137 | 0.135 | -0.092 | 0.019 | -0.078 | 0.058 | 0.005 |
| PC aa C42:4 | 0.089 | 0.030 | 0.019 | 0.027 | 0.031 | -0.019 | -0.085 | 0.137 | -0.074 |
| PC aa C42:5 | 0.142 | 0.040 | -0.029 | -0.022 | -0.176 | 0.007 | -0.037 | 0.152 | -0.023 |
| **Acyl-alkyl-phosphatidylcholines** | | |  |  |  |  |  |  |  |
| PC ae C30:0 | 0.158 | -0.034 | -0.029 | -0.195 | -0.025 | 0.062 | 0.042 | -0.093 | -0.023 |
| PC ae C30:2 | 0.127 | 0.028 | 0.039 | -0.136 | -0.026 | 0.030 | 0.061 | -0.040 | 0.036 |
| PC ae C32:1 | 0.113 | -0.035 | 0.021 | -0.019 | 0.036 | -0.005 | 0.018 | -0.008 | -0.064 |
| PC ae C32:2 | 0.119 | -0.004 | 0.078 | 0.011 | -0.016 | 0.004 | 0.015 | -0.012 | 0.031 |
| PC ae C34:0 | 0.153 | -0.040 | -0.017 | -0.078 | -0.006 | 0.048 | 0.084 | -0.127 | -0.006 |
| PC ae C34:1 | 0.120 | -0.047 | -0.027 | -0.050 | 0.054 | -0.022 | 0.052 | -0.096 | -0.022 |
| PC ae C34:2 | 0.102 | -0.010 | 0.076 | -0.068 | 0.104 | 0.040 | 0.016 | 0.015 | 0.034 |
| PC ae C34:3 | 0.104 | 0.009 | 0.095 | -0.034 | 0.110 | 0.020 | -0.031 | 0.061 | 0.023 |
| PC ae C36:0 | 0.121 | -0.004 | 0.032 | 0.085 | -0.066 | -0.036 | -0.002 | -0.026 | 0.034 |
| PC ae C36:1 | 0.133 | -0.030 | -0.011 | -0.079 | 0.034 | 0.001 | 0.087 | -0.142 | 0.033 |
| PC ae C36:2 | 0.105 | 0.030 | 0.075 | -0.129 | 0.063 | 0.055 | 0.048 | -0.035 | 0.053 |
| PC ae C36:3 | 0.101 | 0.000 | 0.060 | -0.043 | 0.102 | 0.015 | -0.016 | 0.055 | 0.031 |
| PC ae C36:4 | 0.086 | -0.078 | 0.030 | 0.075 | 0.162 | -0.021 | -0.005 | 0.040 | -0.066 |
| PC ae C36:5 | 0.100 | -0.072 | 0.063 | 0.143 | 0.087 | -0.018 | -0.017 | 0.006 | -0.036 |
| PC ae C38:2 | 0.114 | 0.049 | 0.051 | -0.072 | 0.038 | 0.040 | -0.020 | 0.038 | 0.024 |
| PC ae C38:3 | 0.114 | -0.005 | -0.013 | -0.105 | 0.073 | 0.018 | 0.058 | -0.027 | -0.029 |
| PC ae C38:4 | 0.096 | -0.043 | 0.028 | -0.022 | 0.153 | -0.028 | 0.014 | 0.005 | -0.076 |
| PC ae C38:5 | 0.078 | -0.032 | 0.067 | 0.111 | 0.106 | -0.044 | -0.010 | 0.013 | -0.061 |
| PC ae C38:6 | 0.106 | -0.023 | 0.125 | 0.166 | -0.046 | 0.005 | 0.023 | -0.045 | 0.020 |
| PC ae C40:1 | 0.126 | 0.052 | 0.054 | 0.076 | -0.044 | 0.018 | -0.107 | 0.017 | 0.077 |
| PC ae C40:2 | 0.128 | 0.016 | 0.058 | -0.078 | -0.054 | 0.013 | 0.097 | -0.091 | 0.000 |
| PC ae C40:3 | 0.105 | 0.047 | 0.048 | -0.096 | 0.023 | 0.007 | 0.013 | 0.025 | -0.055 |
| PC ae C40:4 | 0.098 | 0.013 | 0.053 | -0.046 | 0.103 | -0.032 | -0.037 | 0.058 | -0.106 |
| PC ae C40:5 | 0.101 | 0.017 | 0.074 | 0.003 | 0.024 | -0.021 | -0.006 | 0.009 | -0.078 |
| PC ae C40:6 | 0.107 | 0.038 | 0.144 | 0.050 | -0.077 | 0.018 | 0.047 | -0.068 | 0.007 |
| Continues | | | | | | | | | |
| **Table S7 continued** | | | | | | | | | |
| **Metabolites** | **PC1** | **PC2** | **PC3** | **PC4** | **PC5** | **PC6** | **PC7** | **PC8** | **PC9** |
| PC ae C42:1 | 0.102 | 0.036 | 0.019 | 0.042 | 0.023 | -0.023 | -0.117 | 0.108 | -0.026 |
| PC ae C42:2 | 0.132 | 0.043 | 0.029 | -0.004 | -0.068 | -0.005 | -0.015 | -0.007 | 0.001 |
| PC ae C42:3 | 0.108 | 0.073 | 0.093 | 0.030 | -0.047 | 0.004 | -0.051 | 0.008 | -0.019 |
| PC ae C42:4 | 0.111 | 0.053 | 0.091 | -0.084 | 0.110 | -0.007 | -0.075 | 0.096 | -0.211 |
| PC ae C42:5 | 0.075 | 0.033 | 0.088 | -0.005 | 0.051 | -0.041 | -0.034 | 0.037 | -0.139 |
| PC ae C44:4 | 0.085 | 0.029 | 0.048 | -0.031 | 0.090 | -0.025 | -0.061 | 0.014 | -0.211 |
| PC ae C44:5 | 0.074 | -0.001 | 0.084 | 0.057 | 0.121 | -0.077 | -0.040 | -0.026 | -0.222 |
| PC ae C44:6 | 0.068 | 0.074 | 0.151 | 0.043 | 0.061 | -0.018 | -0.085 | 0.090 | -0.220 |
| **HEXOSE** |  |  |  |  |  |  |  |  |  |
| Hexose | -0.006 | -0.255 | 0.060 | 0.060 | 0.027 | 0.049 | 0.063 | 0.126 | 0.043 |
| **SPHINGOLIPIDS** |  |  |  |  |  |  |  |  |  |
| **Hydroxysphingomyelins** | |  |  |  |  |  |  |  |  |
| SM (OH) C14:1 | 0.097 | 0.018 | 0.062 | -0.163 | 0.044 | 0.039 | 0.163 | -0.132 | 0.001 |
| SM (OH) C16:1 | 0.076 | 0.001 | 0.100 | -0.102 | 0.069 | 0.020 | 0.195 | -0.168 | -0.012 |
| SM (OH) C22:1 | 0.061 | -0.002 | 0.065 | -0.015 | 0.083 | 0.010 | 0.139 | -0.127 | -0.056 |
| SM (OH) C22:2 | 0.082 | 0.022 | 0.081 | -0.084 | 0.035 | 0.001 | 0.132 | -0.137 | 0.006 |
| SM (OH) C24:1 | 0.049 | -0.013 | 0.085 | 0.009 | 0.108 | 0.006 | 0.144 | -0.162 | -0.114 |
| **Sphingomyelins** |  |  |  |  |  |  |  |  |  |
| SM C16:0 | 0.057 | 0.010 | 0.077 | -0.001 | 0.062 | -0.025 | 0.084 | -0.021 | -0.033 |
| SM C16:1 | 0.063 | 0.008 | 0.048 | -0.009 | 0.053 | -0.035 | 0.077 | 0.005 | -0.004 |
| SM C18:0 | 0.065 | -0.045 | 0.030 | 0.002 | 0.051 | -0.030 | 0.166 | -0.088 | -0.059 |
| SM C18:1 | 0.062 | -0.005 | 0.046 | -0.019 | 0.048 | -0.040 | 0.144 | -0.052 | -0.013 |
| SM C20:2 | 0.105 | 0.168 | 0.097 | -0.366 | -0.090 | -0.042 | 0.147 | 0.408 | 0.257 |
| SM C24:0 | 0.049 | -0.006 | 0.031 | 0.060 | 0.076 | -0.010 | 0.071 | -0.009 | -0.097 |
| SM C24:1 | 0.056 | 0.002 | 0.094 | 0.075 | 0.020 | -0.063 | 0.086 | -0.058 | -0.039 |
| Variance explained, % | 24.4 | 9.5 | 7.3 | 6.1 | 5.6 | 5.0 | 4.2 | 3.0 | 2.7 |

Abbreviations: EPIC, European Prospective Investigation into Cancer and Nutrition; PC, principal component.
Red and yellow highlight loadings higher than 0.1 and lower than -0.1, respectively.

## Table S8. Correlations between scores for metabolite patterns derived using treelet transform and principal component analysis in 3057 control participants from EPIC

|  | **TC1** | **TC2** | **TC3** |
| --- | --- | --- | --- |
| **PC1** | 0.99 | -0.03 | 0.54 |
| **PC2** | -0.06 | 0.88 | 0.27 |
| **PC3** | <0.01 | -0.30 | -0.14 |
| **PC4** | 0.03 | 0.19 | 0.10 |
| **PC5** | -0.08 | <0.01 | 0.43 |
| **PC6** | 0.03 | <0.01 | -0.12 |
| **PC7** | 0.01 | 0.13 | -0.40 |
| **PC8** | 0.01 | 0.03 | -0.21 |
| **PC9** | -0.05 | -0.13 | 0.38 |

Abbreviations: EPIC, European Prospective Investigation into Cancer and Nutrition; PC, principal component; TC, Treelet component

**Table S9. Risk of prostate cancer in relation to principal component scores in 3057 matched case-control sets from EPIC1**

|  | **n_case_** | **1 standard deviation increase** | | |
| --- | --- | --- | --- | --- |
|  |  | **OR (95% CI)** | **p** | **p-het** |
| **PC1** |  |  |  |  |
| Overall prostate cancer | 3057 | 0.94 (0.88 to 1.00) | 0.049 |  |
| Stage |  |  |  |  |
| Localised^2^ | 1306 | 1.02 (0.93 to 1.13) | 0.648 |  |
| Advanced^3^ | 580 | 0.76 (0.66 to 0.89) | 0.001 | 0.001 |
| Follow-up ≤10 yrs | 328 | 0.76 (0.62 to 0.93) | 0.009 |  |
| Follow-up >10 yrs | 252 | 0.78 (0.61 to 0.99) | 0.038 | 0.894 |
| Non-aggressive^4^ | 1519 | 0.98 (0.90 to 1.07) | 0.669 |  |
| Aggressive^5^ | 367 | 0.79 (0.65 to 0.94) | 0.010 | 0.014 |
| Follow-up ≤10 yrs | 229 | 0.76 (0.60 to 0.96) | 0.020 |  |
| Follow-up >10 yrs^6^ | 137 | 0.83 (0.61 to 1.14) | 0.244 | 0.730 |
| Grade |  |  |  |  |
| Low-intermediate^7^ | 2157 | 0.95 (0.88 to 1.03) | 0.205 |  |
| High^8^ | 317 | 0.87 (0.71 to 1.07) | 0.195 | 0.593 |
| Prostate cancer death^9^ | 297 | 0.96 (0.79 to 1.18) | 0.711 |  |
|  |  |  |  |  |
| **PC2** |  |  |  |  |
| Overall prostate cancer | 3057 | 0.99 (0.90 to 1.09) | 0.861 |  |
| Stage |  |  |  |  |
| Localised^2^ | 1306 | 1.09 (0.94 to 1.27) | 0.243 |  |
| Advanced^3^ | 580 | 0.73 (0.57 to 0.93) | 0.010 | 0.001 |
| Follow-up ≤10 yrs | 328 | 0.73 (0.53 to 1.00) | 0.051 |  |
| Follow-up >10 yrs | 252 | 0.75 (0.50 to 1.11) | 0.145 | 0.838 |
| Non-aggressive^4^ | 1519 | 1.04 (0.91 to 1.20) | 0.551 |  |
| Aggressive^5^ | 367 | 0.71 (0.52 to 0.97) | 0.030 | 0.006 |
| Follow-up ≤10 yrs | 229 | 0.63 (0.42 to 0.95) | 0.028 |  |
| Follow-up >10 yrs^6^ | 137 | 0.83 (0.50 to 1.39) | 0.485 | 0.303 |
| Grade |  |  |  |  |
| Low-intermediate^7^ | 2157 | 0.97 (0.86 to 1.08) | 0.574 |  |
| High^8^ | 317 | 0.94 (0.69 to 1.27) | 0.670 | 0.588 |
| Prostate cancer death^9^ | 297 | 0.88 (0.65 to 1.18) | 0.379 |  |
|  |  |  |  |  |
| **PC3** |  |  |  |  |
| Overall prostate cancer | 3057 | 1.05 (0.98 to 1.11) | 0.152 |  |
| Stage |  |  |  |  |
| Localised^2^ | 1306 | 1.07 (0.98 to 1.18) | 0.139 |  |
| Advanced^3^ | 580 | 1.06 (0.91 to 1.23) | 0.474 | 0.261 |
| Follow-up ≤10 yrs | 328 | 1.01 (0.81 to 1.26) | 0.920 |  |
| Follow-up >10 yrs | 252 | 1.12 (0.89 to 1.40) | 0.341 | 0.211 |
| Non-aggressive^4^ | 1519 | 1.07 (0.98 to 1.17) | 0.126 |  |
| Aggressive^5^ | 367 | 1.05 (0.86 to 1.27) | 0.646 | 0.305 |
| Follow-up ≤10 yrs | 229 | 1.03 (0.79 to 1.35) | 0.826 |  |
| Follow-up >10 yrs^6^ | 137 | 1.03 (0.75 to 1.41) | 0.867 | 0.840 |
| Grade |  |  |  |  |
| Low-intermediate^7^ | 2157 | 1.07 (1.00 to 1.16) | 0.062 |  |
| High^8^ | 317 | 0.92 (0.75 to 1.12) | 0.390 | 0.050 |
| Prostate cancer death^9^ | 297 | 0.99 (0.78 to 1.24) | 0.906 |  |
| Continues |  |  |  |  |

Table S9 continued

|  | **n_case_** | **1 standard deviation increase** | | |
| --- | --- | --- | --- | --- |
|  |  | **OR (95% CI)** | **p** | **p-het** |
| **PC4** |  |  |  |  |
| Overall prostate cancer | 3057 | 0.98 (0.92 to 1.05) | 0.636 |  |
| Stage |  |  |  |  |
| Localised^2^ | 1306 | 1.07 (0.96 to 1.19) | 0.225 |  |
| Advanced^3^ | 580 | 0.80 (0.68 to 0.94) | 0.008 | 0.050 |
| Follow-up ≤10 yrs | 328 | 0.82 (0.66 to 1.03) | 0.084 |  |
| Follow-up >10 yrs | 252 | 0.76 (0.59 to 0.98) | 0.038 | 0.427 |
| Non-aggressive^4^ | 1519 | 1.06 (0.96 to 1.17) | 0.244 |  |
| Aggressive^5^ | 367 | 0.73 (0.59 to 0.90) | 0.003 | 0.005 |
| Follow-up ≤10 yrs | 229 | 0.84 (0.65 to 1.09) | 0.193 |  |
| Follow-up >10 yrs^6^ | 137 | 0.54 (0.37 to 0.80) | 0.002 | 0.150 |
| Grade |  |  |  |  |
| Low-intermediate^7^ | 2157 | 0.98 (0.90 to 1.07) | 0.680 |  |
| High^8^ | 317 | 0.76 (0.61 to 0.96) | 0.021 | 0.571 |
| Prostate cancer death^9^ | 297 | 1.17 (0.92 to 1.49) | 0.193 |  |
|  |  |  |  |  |
| **PC5** |  |  |  |  |
| Overall prostate cancer | 3057 | 0.97 (0.91 to 1.03) | 0.275 |  |
| Stage |  |  |  |  |
| Localised^2^ | 1306 | 0.92 (0.84 to 1.01) | 0.095 |  |
| Advanced^3^ | 580 | 1.00 (0.87 to 1.15) | 0.968 | 0.659 |
| Follow-up ≤10 yrs | 328 | 1.02 (0.84 to 1.25) | 0.808 |  |
| Follow-up >10 yrs | 252 | 0.98 (0.80 to 1.22) | 0.879 | 0.879 |
| Non-aggressive^4^ | 1519 | 0.94 (0.87 to 1.03) | 0.191 |  |
| Aggressive^5^ | 367 | 0.97 (0.81 to 1.16) | 0.720 | 0.823 |
| Follow-up ≤10 yrs | 229 | 0.88 (0.69 to 1.12) | 0.312 |  |
| Follow-up >10 yrs^6^ | 137 | 1.16 (0.87 to 1.55) | 0.316 | 0.218 |
| Grade |  |  |  |  |
| Low-intermediate^7^ | 2157 | 0.96 (0.89 to 1.03) | 0.247 |  |
| High^8^ | 317 | 1.08 (0.89 to 1.30) | 0.457 | 0.347 |
| Prostate cancer death^9^ | 297 | 0.97 (0.79 to 1.19) | 0.768 |  |
|  |  |  |  |  |
| **PC6** |  |  |  |  |
| Overall prostate cancer | 3057 | 1.05 (0.99 to 1.11) | 0.082 |  |
| Stage |  |  |  |  |
| Localised^2^ | 1306 | 1.05 (0.96 to 1.14) | 0.278 |  |
| Advanced^3^ | 580 | 1.06 (0.94 to 1.21) | 0.330 | 0.758 |
| Follow-up ≤10 yrs | 328 | 1.04 (0.88 to 1.23) | 0.682 |  |
| Follow-up >10 yrs | 252 | 1.09 (0.90 to 1.33) | 0.385 | 0.780 |
| Non-aggressive^4^ | 1519 | 1.06 (0.98 to 1.14) | 0.163 |  |
| Aggressive^5^ | 367 | 1.04 (0.89 to 1.22) | 0.598 | 0.792 |
| Follow-up ≤10 yrs | 229 | 1.02 (0.85 to 1.24) | 0.814 |  |
| Follow-up >10 yrs^6^ | 137 | 1.06 (0.80 to 1.41) | 0.681 | 0.645 |
| Grade |  |  |  |  |
| Low-intermediate^7^ | 2157 | 1.04 (0.97 to 1.11) | 0.265 |  |
| High^8^ | 317 | 1.02 (0.86 to 1.22) | 0.819 | 0.933 |
| Prostate cancer death^9^ | 297 | 1.09 (0.92 to 1.29) | 0.322 |  |
| Continues |  |  |  |  |

Table S9 continued

|  | **n_case_** | **1 standard deviation increase** | | |
| --- | --- | --- | --- | --- |
|  |  | **OR (95% CI)** | **p** | **p-het** |
| **PC7** |  |  |  |  |
| Overall prostate cancer | 3057 | 1.02 (0.96 to 1.08) | 0.574 |  |
| Stage |  |  |  |  |
| Localised^2^ | 1306 | 1.01 (0.91 to 1.11) | 0.917 |  |
| Advanced^3^ | 580 | 1.02 (0.88 to 1.18) | 0.797 | 0.603 |
| Follow-up ≤10 yrs | 328 | 0.96 (0.79 to 1.17) | 0.662 |  |
| Follow-up >10 yrs | 252 | 1.14 (0.90 to 1.44) | 0.276 | 0.441 |
| Non-aggressive^4^ | 1519 | 1.00 (0.92 to 1.10) | 0.920 |  |
| Aggressive^5^ | 367 | 1.03 (0.86 to 1.24) | 0.718 | 0.447 |
| Follow-up ≤10 yrs | 229 | 1.01 (0.81 to 1.26) | 0.915 |  |
| Follow-up >10 yrs^6^ | 137 | 1.07 (0.77 to 1.49) | 0.684 | 0.951 |
| Grade |  |  |  |  |
| Low-intermediate^7^ | 2157 | 0.99 (0.92 to 1.06) | 0.751 |  |
| High^8^ | 317 | 1.03 (0.84 to 1.27) | 0.762 | 0.635 |
| Prostate cancer death^9^ | 297 | 1.32 (1.06 to 1.64) | 0.014 |  |
|  |  |  |  |  |
| **PC8** |  |  |  |  |
| Overall prostate cancer | 3057 | 0.91 (0.86 to 0.97) | 0.002 |  |
| Stage |  |  |  |  |
| Localised^2^ | 1306 | 0.90 (0.83 to 0.98) | 0.022 |  |
| Advanced^3^ | 580 | 0.84 (0.74 to 0.96) | 0.011 | 0.825 |
| Follow-up ≤10 yrs | 328 | 0.81 (0.67 to 0.97) | 0.020 |  |
| Follow-up >10 yrs | 252 | 0.86 (0.70 to 1.06) | 0.161 | 0.839 |
| Non-aggressive^4^ | 1519 | 0.92 (0.84 to 0.99) | 0.036 |  |
| Aggressive^5^ | 367 | 0.76 (0.65 to 0.90) | 0.002 | 0.234 |
| Follow-up ≤10 yrs | 229 | 0.78 (0.63 to 0.96) | 0.020 |  |
| Follow-up >10 yrs^6^ | 137 | 0.67 (0.50 to 0.92) | 0.012 | 0.575 |
| Grade |  |  |  |  |
| Low-intermediate^7^ | 2157 | 0.93 (0.87 to 0.99) | 0.027 |  |
| High^8^ | 317 | 0.81 (0.67 to 0.99) | 0.036 | 0.616 |
| Prostate cancer death^9^ | 297 | 1.05 (0.87 to 1.26) | 0.626 |  |
|  |  |  |  |  |
| **PC9** |  |  |  |  |
| Overall prostate cancer | 3057 | 0.98 (0.93 to 1.04) | 0.552 |  |
| Stage |  |  |  |  |
| Localised^2^ | 1306 | 1.01 (0.93 to 1.10) | 0.822 |  |
| Advanced^3^ | 580 | 0.95 (0.83 to 1.10) | 0.502 | 0.408 |
| Follow-up ≤10 yrs | 328 | 0.95 (0.79 to 1.15) | 0.604 |  |
| Follow-up >10 yrs | 252 | 0.97 (0.78 to 1.21) | 0.778 | 0.738 |
| Non-aggressive^4^ | 1519 | 1.00 (0.93 to 1.09) | 0.913 |  |
| Aggressive^5^ | 367 | 0.94 (0.79 to 1.11) | 0.462 | 0.390 |
| Follow-up ≤10 yrs | 229 | 0.96 (0.77 to 1.20) | 0.736 |  |
| Follow-up >10 yrs^6^ | 137 | 0.93 (0.69 to 1.25) | 0.614 | 0.861 |
| Grade |  |  |  |  |
| Low-intermediate^7^ | 2157 | 0.99 (0.93 to 1.06) | 0.834 |  |
| High^8^ | 317 | 0.91 (0.75 to 1.10) | 0.308 | 0.704 |
| Prostate cancer death^9^ | 297 | 0.85 (0.70 to 1.03) | 0.095 |  |

Abbreviations: EPIC, European Prospective Investigation into Cancer and Nutrition; OR, odds ratio; CI confidence interval; p-het, p value for heterogeneity; TC, treelet component; yrs, years.

^1^ All analyses were matched on centre, length of follow-up, and age (±6 months), time of day (±1 hour) and fasting status (<3, 3–6, >6 hours) at blood collection and further adjusted for exact age at blood collection (continuously), and baseline values for body mass index (quartiles; unknown), smoking (never; past; current; unknown), alcohol intake (<10; 10-19; 20-39; ≥40 g of alcohol per day; unknown), education (primary; secondary; degree level; unknown) and marital status (married or cohabiting; not married or cohabiting; unknown).

^2^ Tumour-node-metastasis score ≤T2 and N0/x and M0, or coded as localised.

^3^ Tumour-node-metastasis score T3-4 and/or N1-3 and/or M1, or coded as advanced.

^4^ Tumour-node-metastasis score ≤T3 and N0/x and M0.

^5^ Tumour-node-metastasis score T4 and/or N1-3 and/or M1.

^6^ One matched set was excluded from this analysis; the case was the only individual with unknown smoking status in this sub-group and the model was thus not stable.

^7^ Gleason score <8 or coded as well, moderately or poorly differentiated.

^8^ Gleason score ≥8 or coded as undifferentiated.

^9^ Matched sets in which the control died, emigrated or was lost to follow-up before the case died were excluded (n=29).

## Reference

1. Schmidt JA, Fensom GK, Rinaldi S, Scalbert A, Appleby PN, Achaintre D, Gicquiau A, Gunter MJ, Ferrari P, Kaaks R, Kuhn T, Floegel A, Boeing H, Trichopoulou A, Lagiou P, Anifantis E, Agnoli C, Palli D, Trevisan M, Tumino R, Bueno-de-Mesquita HB, Agudo A, Larranaga N, Redondo-Sanchez D, Barricarte A, Huerta JM, Quiros JR, Wareham N, Khaw KT, Perez-Cornago A, Johansson M, Cross AJ, Tsilidis KK, Riboli E, Key TJ, Travis RC. Pre-diagnostic metabolite concentrations and prostate cancer risk in 1077 cases and 1077 matched controls in the European Prospective Investigation into Cancer and Nutrition. BMC Med 2017; 15: 122
